# Cytokinetic bridge triggers de novo lumen formation in vivo

**DOI:** 10.1038/s41467-020-15002-8

**Published:** 2020-03-09

**Authors:** L. I. Rathbun, E. G. Colicino, J. Manikas, J. O’Connell, N. Krishnan, N. S. Reilly, S. Coyne, G. Erdemci-Tandogan, A. Garrastegui, J. Freshour, P. Santra, M. L. Manning, J. D. Amack, H. Hehnly

**Affiliations:** 1 Biology Department, Syracuse University, Syracuse, New York USA; 2 Department of Cell and Developmental Biology, SUNY Upstate Medical School, Syracuse, New York USA; 3 Department of Physics and Astronomy, University of Rochester, Rochester, New York USA; 4 Department of Biology, SUNY Geneseo, Geneseo, New York USA; 5 Department of Physics, Syracuse University, Syracuse, New York USA; 6 Present Address: Department of Cell and Developmental Biology, University of Michigan Medical School, Ann Arbor, Michigan USA

**Keywords:** Cell division, Cell polarity, Organogenesis

## Abstract

Multicellular rosettes are transient epithelial structures that serve as intermediates during diverse organ formation. We have identified a unique contributor to rosette formation in zebrafish Kupffer’s vesicle (KV) that requires cell division, specifically the final stage of mitosis termed abscission. KV utilizes a rosette as a prerequisite before forming a lumen surrounded by ciliated epithelial cells. Our studies identify that KV-destined cells remain interconnected by cytokinetic bridges that position at the rosette’s center. These bridges act as a landmark for directed Rab11 vesicle motility to deliver an essential cargo for lumen formation, CFTR (cystic fibrosis transmembrane conductance regulator). Here we report that premature bridge cleavage through laser ablation or inhibiting abscission using optogenetic clustering of Rab11 result in disrupted lumen formation. We present a model in which KV mitotic cells strategically place their cytokinetic bridges at the rosette center, where Rab11-associated vesicles transport CFTR to aid in lumen establishment.

## Abstract (web summary)

De novo lumen formation during vertebrate left–right organizer development is required for body axis establishment. Here the authors utilize zebrafish to demonstrate that the position and cleavage of the cytokinetic bridge in dividing left-right organizer cells dictates tissue morphogenesis.

## Introduction

Tissue morphogenesis is a fundamental process that contributes to building and maintaining organs, as well as orchestrating overall embryogenesis^1^. How these morphogenic changes are coordinated at a molecular and cellular level remains a central question to developmental biology. One common cellular rearrangement that occurs during tissue morphogenesis is the generation of a transient epithelial rosette that remodels to form a finalized organ with apical-basal polarity and a central lumen. Rosettes are multicellular structures that interface at a central point. Rosette formation has been observed in many contexts including *Drosophila* eye morphogenesis, zebrafish lateral line development, mouse and *Xenopus* kidney tubule formation, and pancreatic branching in mice^2–5^. Our studies here utilize the left–right organizer, Kupffer’s vesicle (KV), in the vertebrate model *Danio rerio* to characterize a mechanism of rosette and subsequent lumen formation.

KV is a conserved organ of asymmetry that is required in all vertebrates to place visceral and abdominal organs with respect to the two main body axes and requires the formation of a rosette structure before it fully develops^6,7^. The mechanism of asymmetry establishment in some mammals (humans, mouse, and rabbit), fish, and amphibians is that the organ of asymmetry creates a leftward flow through motile cilia in the extracellular lumen to initiate the asymmetrical expression of three genes, *Nodal*, *Lefty*, and *Pitx2*, across the embryo^8^. Due to the conservation of this organ, the ease of transgenics, and live-cell imaging of a transparent embryo, KV was used as an in vivo model for lumen formation. The current framework for KV development is that non-polarized mesenchymal cells organize into a two-dimensional rosette-like structure that will assemble into a three-dimensional (3D) sphere with a fluid-filled lumen. During rosette assembly, the individual cells start to establish apicobasal polarity^9^. Although events downstream of KV’s leftward fluid flow have received much attention^10^, little is known about the mechanism required for KV assembly.

KV rosette formation may require the actin–myosin network at the apical membrane^9^. This same actin–myosin network drives contractile ring formation during cytokinesis, a process of separating the two daughter cells following mitosis^11^. Following cytokinesis, a cytokinetic bridge remains between the two daughter cells for a duration of 1–3 h depending on cell type^12,13^. The remaining bridge is cleaved in a process called abscission. To accomplish this, the bridge is first resolved to a diameter of ~1–2 μm permitting the Endosomal Sorting Complexes Required for Transport to sever the bridge^14,15^. In 3D kidney epithelial cell cultures that form a sphere with a central lumen, the cytokinetic bridge furrow ingression occurs towards the center of the sphere where the apical membrane is established^16,17^.

Here, our studies demonstrate that the cytokinetic bridge acts as a symmetry-breaking event to signal where the apical membrane of the dividing cell is positioned. We find in the developing zebrafish embryo that the process of cell division is required for KV morphogenesis. Specifically, the placement of the cytokinetic bridge and its appropriate cleavage is involved in KV transition from a rosette structure to a sphere with a fluid-filled lumen.

## Results and discussion

### Mitosis is required for lumen formation

**Fig. 1:**
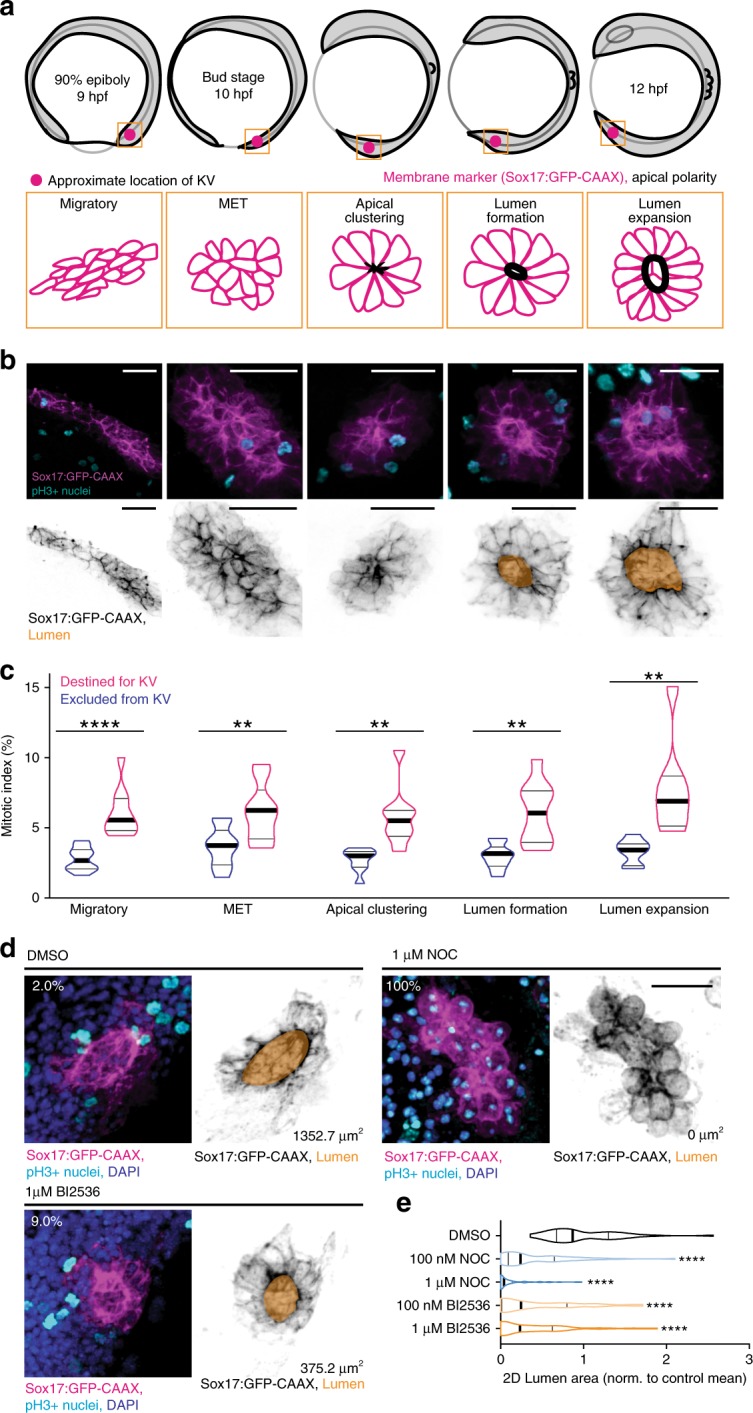
Mitosis is required for lumen formation. **a** Model depicting zebrafish embryo (top) and KV morphology (bottom) during development. Approximate location of KV denoted by magenta spot. KV membrane (magenta) and regions of apical polarity (black) depicted in model below. **b** Top: maximum confocal projections of KV at developmental stages denoted in **a**. pH3 (mitotic nuclei, cyan) and KV membrane marker (Sox17:GFP-CAAX, magenta) shown. Bottom: KV membrane marker (Sox17:GFP-CAAX—gray) and lumen trace (orange) shown. Bars, 50 μm. **c** Mitotic indices (%) represented as violin plot with endpoints depicting minimum and maximum values, quartiles depicted by thin black lines, median depicted by thick black line. *n* > 247 cells/stage, *n* = 43 embryos, two-tailed, unpaired Student’s *t*-test. Statistical results detailed in Supplementary Table 5. **d** Representative 3D renderings of KV under conditions of DMSO vehicle control, microtubule inhibition (1 μM nocodazole), or PLK1 inhibition (1 μM BI2536). Sox17:GFP-CAAX (magenta), pH3-positive nuclei (cyan), and DAPI (blue) shown on the left. Sox17:GFP-CAAX (gray) and lumen trace (orange) shown on the right. Percentages indicate mitotic index of image, lumen area denoted. Bar, 20 μm. **e** Violin plot depicting normalized 2D lumen area under conditions represented in **d** with endpoints depicting minimum and maximum values, quartiles depicted by thin black lines, and median depicted by thick black line. One-way ANOVA with Dunnett’s multiple comparison and statistical results are detailed in Supplementary Table 5 (****p < 0.0001 for *n* > 41 embryos).

KV uses a rosette intermediate before forming a lumen (Fig. 1a, b)^9^. KV precursor cells are visualized in live embryos by decorating the plasma membrane with green fluorescent protein (GFP) (Sox17:GFP-CAAX; Fig. 1b). The current framework for KV development is that mesenchymal precursor cells transition to epithelial KV cells (mesenchymal-epithelial transition, MET), which requires establishment of apicobasal polarity, apical clustering, and the expansion of apical cell surfaces to facilitate the formation of a central lumen^18^ (Fig. 1a, b). To investigate the contribution of cell division to KV development, we first calculated the mitotic index of cells destined to form KV compared with other stages of development. The mitotic index was measured during the first 24 h post fertilization (hpf). In this time frame, embryos transition through four basic developmental stages: the cleavage period (0–2.25 hpf), the blastula period (2.25–5.25 hpf), the gastrula period (5.25–10 hpf), and the segmentation period^19^ (10–24 hpf; Supplementary Fig. 1a). KV formation occurs between the gastrula and segmentation period. During the cleavage period, the mitotic index is 100% and steadily decreases to ~3% during the subsequent periods (Supplementary Fig. 1a-c and Supplementary Movie 1). Between the gastrula and segmentation period, KV cells had a mitotic index between 5% and 10% as seen by pH3-positive cells in fixed embryos (Fig. 1b, c) or with PLK1-mCherry in live embryos (Polo-like Kinase 1 (PLK1); Supplementary Movies 2–4). This index was significantly greater than the mitotic index in cells outside the KV (Fig. 1c), suggesting division entry is upregulated in KV-destined cells, providing a program where division is incorporated to contribute to KV morphogenesis.

To determine whether cell division is required for KV lumen formation, we treated cells with two different mitotic synchronizing agents: a small molecule inhibitor of an essential mitotic kinase, PLK1 (BI2536, used in refs. ^20,21^), or a low dose of a microtubule-destabilizing drug to disrupt spindle dynamics (nocodazole, used in ref. ^22^). PLK1 inhibition can result in cells arresting in G2, prometaphase, metaphase, or cytokinesis^23^. Nocodazole treatment can cause overall microtubule destabilization at high doses leading to disruption of intracellular trafficking (usually at 10 μM)^24,25^, but at lower doses (100 nM) the majority of microtubules are intact and defects in prometaphase exit occur^20,23,26^. During 75–90% epiboly, dechorionated embryos were treated with a vehicle control (dimethyl sulfoxide (DMSO)), nocodazole (100 nM or 1 μM), or BI2536 (100 nM or 1 μM; Fig. 1d and Supplementary Fig. 1d). The embryos were allowed to develop to a six-somite stage, where control embryos had a fully developed lumen (Fig. 1d, e). However, the lumen area was significantly lower after BI2536 or nocodazole treatments (Fig. 1d, e). BI2536 and nocodazole treatments resulted in significantly increased mitotic indices compared with control DMSO-treated embryos (Supplementary Fig. 1d–e) and significant decreases in KV cell number (Supplementary Fig. 1f), suggesting that defects in cell proliferation resulted in abnormal lumens. When comparing the lumen area and number of KV cells for each embryo analyzed, a positive relationship between these two variables occurred such that an increase in the KV cell number correlates with an increase in the KV lumen area (Supplementary Fig. 1g). These studies suggest that defects in lumen formation occur when cell division is disrupted.

### Cytokinetic midbodies localize to apical membranes

We hypothesized that KV cell placement post division may be driving KV development and therefore needed to establish both live and fixed markers of mitotic machinery during zebrafish embryo development. In addition to the nuclear marker H2B-Dendra that marks all nuclei (Supplementary Fig. 2a and Supplementary Movie 1), two markers were developed for live-cell microscopy in zebrafish: PLK1 (PLK1-mCherry^20^; Supplementary Fig. 2b) and/or mitotic kinesin-like protein (GFP/mKate-MKLP1; Supplementary Fig. 2c–d and Supplementary Movies 2–4). PLK1 is an essential mitotic kinase that localizes to mitotic spindle poles, kinetochores, and cytokinetic midbodies in dividing cells^23^. Following division, the separation of two daughter cells occurs through the ingression of a cleavage furrow. A complex containing MKLP1 and RacGAP, called centralspindlin, contributes to cleavage furrow ingression^27^. After furrow ingression, dividing animal cells stay interconnected by a narrow intercellular bridge that contains a proteinaceous structure known as the midbody, containing RacGAP and MKLP1 (Supplementary Fig. 2e–i). Although daughter cells remain interconnected, the PLK1-positive centrosomes stay on the far side of the nucleus in relation to the cytokinetic bridge and associated midbody (Supplementary Fig. 2b–e). These studies demonstrate that PLK1-mCherry and GFP/mKate-MKLP1 can be used for monitoring cell cycle progression in vivo due to their similar localization patterns as in in-vitro contexts^20,26,28^.

**Fig. 2:**
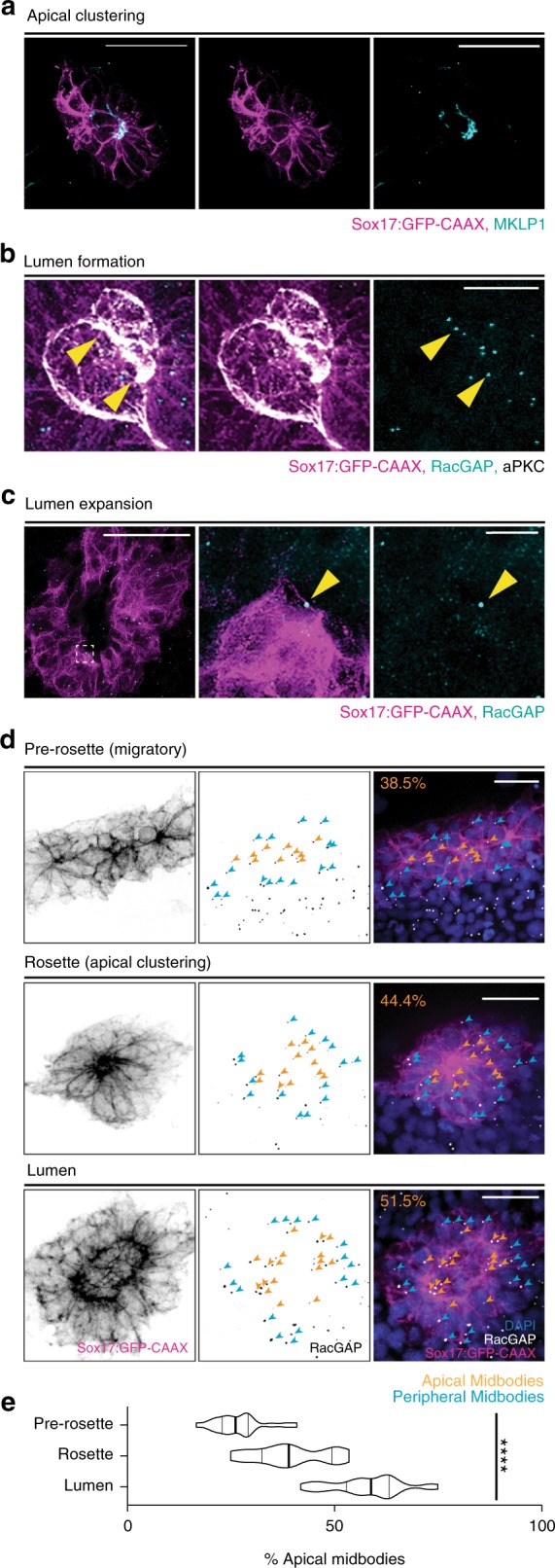
Cytokinetic midbodies localize to apical membranes of lumens in vivo. **a**–**c** Maximum confocal projections of KV in zebrafish embryos during apical clustering (**a**), lumen formation (**b**), and lumen expansion (**c**). Immunolabeled for midbodies (MKLP1 (**a**) or RacGAP (**b**, **c**)—cyan), a polarity marker (aPKC (**b**)—white), and a membrane marker (Sox17:GFP-CAAX—magenta). Bars, 50 μm (**a**, **c**), 20 μm (**b**), and 10 μm (**c** inset). Midbodies localizing to apical membrane during KV lumen formation and lumen expansion denoted by yellow arrowheads (**b**, **c**). **d** Representative images of midbody localization (RacGAP—white) within KV (Sox17:GFP-CAAX—magenta and DAPI—blue). Pre-rosette (top), rosette (middle), and lumen (bottom) stages of KV development depicted. Orange arrowheads denote apical midbodies; cyan arrowheads denote peripheral midbodies. Bar, 50 μm. **e** Violin plot depicting percentage of apical midbodies in KVs at pre-rosette (*n* = 21 embryos), rosette (*n* = 16 embryos), and lumen (*n* = 35 embryos) stages. Endpoints depict minimum and maximum values, quartiles depicted by thin black lines, median depicted by thick black line. *n* > 4 independent experiments. One-way ANOVA, ****p < 0.0001, F(2,69) = 104.7, df = 69.

During zebrafish apical clustering, endogenous MKLP1 is enriched at sites where apical membranes are initiated (as shown by antibody staining in Fig. 2a). In KVs with newly initiated lumens, RacGAP-positive midbodies organize at the apical membrane (decorated with aPKC, atypical protein kinase C; Fig. 2b). With stimulated emission depletion (STED) microscopy, we noted aPKC localizing to the cytokinetic bridge adjacent to the midbody (positive for RacGAP and the bridge positive for acetylated microtubules; Supplementary Fig. 2h). Midbodies were also noted in the newly formed lumen still connected to the apical membrane (using KV membrane marker GFP-CAAX; Supplementary Fig. 2f). During KV lumen expansion, cytokinetic bridges are located closest to the lumen edge and have an associated midbody (Fig. 2c). Midbodies were noted within the lumen surrounded by membrane that were likely released after the bridge was abscised (Supplementary Fig. 2g). To quantify midbody localization throughout KV development, midbodies were scored based on their location, either as apical or peripheral (quantification modeled in Supplementary Fig. 2k). We determined that the percentage of apical midbodies significantly increases as KV develops from pre-rosette to rosette, to lumen stages (Fig. 2d, e). These findings are consistent with an analysis of human fetal tissues where the accumulation of KIF14-positive midbodies were identified in the lumen of ureteric bud tips^29^.

Using an in-vitro 3D epithelial cell model (Madin-Darby canine kidney, MDCK), we noted MKLP1-positive midbodies organizing to the site of apical membrane assembly (Supplementary Fig. 2j). This is consistent with previous in-vitro 3D tissue culture studies demonstrating that the cytokinetic bridge constricts towards the apical lumen^16,30^. This finding corroborates our data in zebrafish embryos during KV apical clustering, lumen formation, and lumen expansion (Fig. 2a–c), and together suggest a role for placement of the cytokinetic bridge in lumen formation.

### Cytokinetic bridges are placed at lumen formation site

**Fig. 3:**
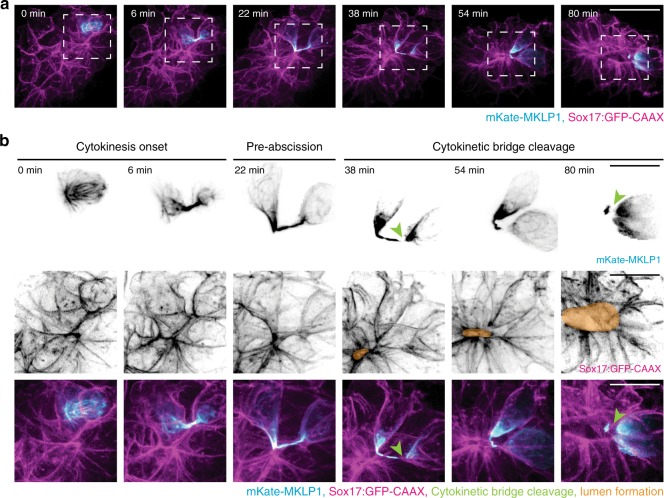
Cytokinetic bridges are placed at the site of future KV lumen formation. **a** A 3D rendering of a cell (mKate-MKLP1, cyan) dividing within KV (Sox17:GFP-CAAX, magenta) over time. Bar, 20 μm. **b** Cell within KV highlighted during cytokinesis onset (left), pre-abscission (center), and cytokinetic bridge cleavage (right). Region denoted with dashed line in **a** are shown in **b**. mKate-MKLP1 (top) and Sox17:GFP-CAAX (bottom) shown in grayscale and in merge below (mKate-MKLP1 in cyan, Sox17:GFP-CAAX in magenta). Green arrowhead denotes the locations of cytokinetic bridge cleavage; orange regions indicate lumen location. Bars, 10 μm.

To examine whether cytokinetic bridge placement is associated with lumen formation, a developing KV was monitored where the cells expressed mKate-MKLP1 (Fig. 3) or PLK1-mCherry (Supplementary Fig. 3a). Upon examination of a cell exiting mitosis, we note that pinching of the cytokinetic bridge places the two daughter cells such that the cytokinetic bridge is positioned where the lumen will form (Fig. 3, Supplementary Fig. 3a, and Supplementary Movie 5). At this time, the bridge is cleaved at one side of the midbody (Fig. 3b, 38 min), then on the other side (Fig. 3b, 80 min.), depositing the midbody into the lumen (Fig. 3b and Supplementary Movie 6). During pre-abscission (22 min; Fig. 3b), daughter cells are noted to start at >10 μm apart and then move to <5 μm apart (Supplementary Fig. 3b), suggesting that daughter cells remain interconnected to pack next to each other into the forming KV.

To quantify changes in cytokinetic bridge/midbody positioning during KV development, we expressed live midbody markers (mKate/mCherry-MKLP1 or PLK1-mCherry) in zebrafish embryos with a KV marker (Sox17:GFP-CAAX or CFTR-GFP). In the same manner as previously used in fixed embryos (Fig. 2d, e and Supplementary 2k), midbodies were scored based on their location (Supplementary Fig. 2k). We calculated a similar trend in live embryos, where the percentage of apical midbodies increased as embryos progressed through pre-rosette, rosette, and lumen formation stages of KV development (Supplementary Fig. 3c–d).

Although spindle orientation is generally thought to be a deciding factor in the placement of daughter cells post division^11,31,32^, we found that daughter cell positioning continues to change throughout cytokinesis and abscission, suggesting that spindle orientation is not always the deciding factor (Fig. 3a, b and Supplementary Movie 5). To directly test whether spindle orientation is utilized in KV development, spindle orientation was measured during the apical clustering and lumen formation stages (Supplementary Fig. 3e–g). If metaphase spindle positioning was a deciding factor in daughter cell placement as in other in vitro and in vivo contexts^31,33,34^, we would predict a Gaussian distribution of spindle orientation values where the majority of spindles orient at a 90° angle to a line passing through the center of a developing KV lumen (predicted Gaussian drawn in Supplementary Fig. 3g, gray line). However, a random distribution of spindle angles in relation to the center of KV was calculated (Supplementary Fig. 3f–g), suggesting that the placement of the cytokinetic bridge is a driving factor in daughter cell positioning during KV development as opposed to spindle positioning.

### Cytokinetic bridge ablation disrupts lumen formation

**Fig. 4:**
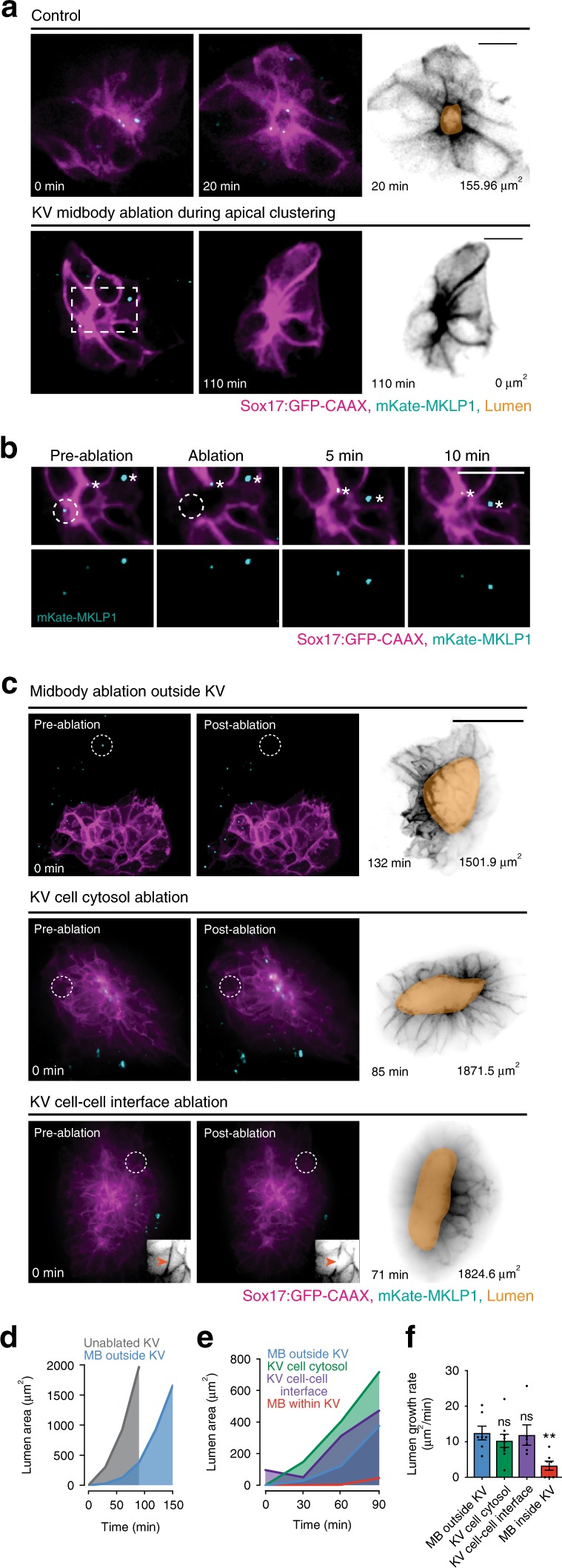
Premature cleavage of the cytokinetic bridge via laser ablation results in disrupted lumen formation. **a** Top: unablated control embryo during apical clustering (left) and lumen formation (center, right). Bottom: central KV midbody ablated during apical clustering from experimental ablation group (left). Subsequent failed lumen formation shown (center, right). KV membrane (Sox17:GFP-CAAX, magenta) and midbody marker (mKate-MKLP1, cyan) shown in the left and center panels; KV membrane (Sox17:GFP-CAAX, gray) and lumen trace (orange) shown on the right. Bar, 20 μm. **b** Midbody ablated in **a**. Pre-ablation, immediately post ablation, and at 5 and 10 min post ablation shown. KV cell membrane (Sox17:GFP-CAAX, magenta) and midbodies (mKate-MKLP1, cyan) shown. Additional unablated midbodies depicted with asterisks. Bar, 10 μm. **c** Representative 3D renderings of KV pre-ablation (left), immediately post ablation (center), and after lumen formation (right) in control groups. Ablation control conditions shown: midbody ablation outside KV (top), KV cell cytosol ablation (middle), KV cell–cell interface ablation (bottom). KV membrane (Sox17:GFP-CAAX—magenta or grayscale), midbodies (mKate-MKLP1—cyan), and lumen trace (orange) shown. Ablation location shown by dotted white circle. Grayscale inset in bottom panel depicts ablation at cell–cell interface within KV (Sox17:GFP-CAAX). **d**, **e** Graphs depicting average lumen area over time for unablated (gray) embryos and embryos with midbody ablated outside KV (blue, **d**), or for embryos with ablation at midbody outside KV (blue, **e**), KV cell cytosol (green, **e**), KV cell membrane (purple, **e**), and midbody within KV (red, **e**). Lumen areas averaged and binned every 30 min. **f** Bar graph depicting rate of lumen area expansion over time. Dots represent individual values. **d**–**f** *n* > 6 embryos/condition across *n* > 3 experiments. ANOVA with Dunnett’s multiple comparison test completed for **d**–**f** compared with embryos with midbody ablated outside KV (blue). Mean displayed ± SEM (**f**). Statistical results detailed in Supplementary Table 5.

As the cytokinetic bridge is placed at the site of future lumen formation, and that from cytokinesis to abscission it takes 1–3 h in vitro^12,13^, we hypothesized that proper spatiotemporal control of abscission is required for lumen formation in KV. To test this idea, we utilized laser ablation to prematurely sever cytokinetic bridges during rosette formation/apical clustering (see Supplementary Fig. 4a for diagram of ablation experiment conditions). In a control embryo, lumen formation begins ~20 min after the apical clustering stage, where cytokinetic midbodies can be seen decorating the site of future lumen formation (Fig. 4a, top). However, when the cytokinetic bridge is prematurely severed through the targeting of a single midbody at the site of lumen formation during apical clustering, lumen formation is either severely diminished or fails altogether (Fig. 4a bottom, 4d–f, and Supplementary Movie 7). Successful ablation was marked by the lack of mKate-MKLP1 fluorescence recovery, with failed midbody ablations resulting in a recovery of midbody fluorescence (Fig. 4b and Supplementary Fig. 4b). An additional example of a successful cytokinetic bridge ablation is under conditions where the cytokinetic bridge is resolved and the section next to the midbody is ablated (Supplementary Fig. 4c). Control ablation experiments were conducted to ensure that lumen formation failure was due to premature bridge severing and not solely to embryo ablation trauma. Control ablation conditions included ablating cytokinetic bridges/midbodies outside KV, ablating KV cell–cell interfaces, and ablating KV cytosol (Fig. 4c–f and modeled in Supplementary Fig. 4a). Although control ablations result in slightly delayed lumen formation compared with unablated controls (Fig. 4d, e), there is no significant difference in lumen formation between control ablation conditions (Fig. 4d–f). However, lumen growth rate was significantly decreased in embryos where a KV cytokinetic bridge was severed during apical clustering compared with control groups (Fig. 4c–f). These experiments suggested that cytokinetic bridges during rosette formation/apical clustering are required for lumen formation.

### Rab11 vesicles are required for abscission in vivo

As premature severing of the cytokinetic bridge perturbed lumen formation, we sought to establish whether blocking abscission altogether would perturb lumen formation as well. Previous work in an in-vitro model has identified that apical-targeted endosomes containing the Par3/aPKC polarity complex assemble adjacent to the cytokinetic midbody^33^. These endosomes contain a small monomeric GTPase, Rab11, required to initiate abscission^35^, making inhibition of the Rab11 vesicle trafficking pathway an ideal method to block abscission in KV development. In zebrafish, Rab11 depletion is associated with KV morphology defects such as lumen size depletion^36,37^. To determine the role of Rab11-associated vesicles in lumen formation and abscission, we acutely inhibited Rab11-associated membrane vesicles through an optogenetic oligomerization approach (modeled from ref. ^38^; Supplementary Fig. 5a).

**Fig. 5:**
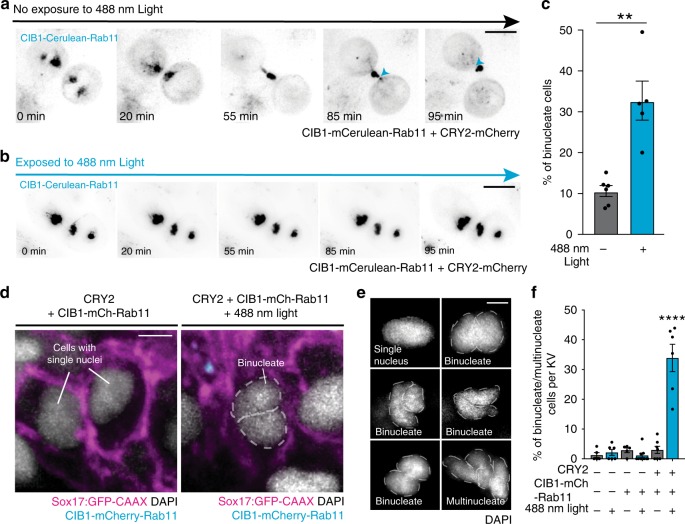
Optogenetic clustering of Rab11-associated vesicles results in failed abscission in vitro and in vivo. **a**, **b** Time-lapse of cytokinetic HeLa cells transfected with CRY2-mCherry and CIB1-mCerulean-Rab11 (black) in the absence (**a**) or presence of 488 nm light (**b**). Bar, 10 μm. Note the cleavage events of cytokinetic bridge (blue arrows, **a**), but not in **b**. **c** Bar graph depicting the percentage of total HeLa cells displaying a binucleate phenotype after being released from a metaphase synchronization for 2 h in the presence or absence of 488 nm light. Cells were transfected with CRY2-mCherry and CIB1-mCerulean-Rab11 as in **a**. Unpaired, two-tailed Mann–Whitney test, **p = 0.0043. Mean displayed ± SEM. *n* = 100 cells per treatment for *n* > 5 experiments. Dots represent individual values. Statistical results detailed in Supplementary Table 5. **d** A 3D rendering of embryos expressing CRY2 and CIB1-mCherry-Rab11 in the absence (left) and presence (right) of 488 nm light. Sox17:GFP-CAAX (magenta), CIB1-mCherry-Rab11 (cyan), and nuclei (DAPI—white) shown. Bar, 5 μm. **e** Representative images of single nuclei, binucleate, or multinucleate cells. Nuclei shown in grayscale (DAPI). Bar, 5 μm. **f** Bar graph depicting percentage of binucleate and/or multinucleate cells per KV in uninjected embryos and embryos expressing CIB1-mCherry-Rab11 or CRY2 and CIB1-mCherry-Rab11 plus or minus 488 nm light. One-way ANOVA with Dunnett’s multiple comparison test used, compared with uninjected embryos in the absence of 488 nm light exposure. Statistical results detailed in Supplementary Table 5. Analyses performed in *n* > 5 embryos over three experiments. Mean displayed ± SEM. Dots represent individual values.

To test the efficacy of this system, we expressed cryptochrome 2-mCherry (CRY2-mCherry) and CIB1-mCerulean-Rab11 in HeLa cells. A blue-light-inducible (488 nm) hetero-interaction between CRY2 and CIB1 is induced within a specific region of interest (ROI), to initiate cellular aggregation of Rab11-associated membranes (Supplementary Fig. 5a–b). To examine whether the cellular aggregation of Rab11-associated membranes disrupts function, HeLa cells expressing the optogenetic constructs in pre-abscission were treated with normal light conditions or 488 nm blue light (Fig. 5a, b). Under control conditions, where cells are imaged in the absence of blue light, cells can progress through cytokinesis to abscission within ~90 min (Fig. 5a). It is noteworthy that CIB1-Cerulean-Rab11 transports into the cytokinetic bridge, where a cleavage event occurs at one side of the midbody (blue arrow; Fig. 5a) and another event occurs on the other side of the midbody 10 min later (blue arrow, Fig. 5a). This is consistent with the events we find in KV with cytokinetic bridge cleavage during lumen formation (Fig. 3b). When cells are exposed to 488 nm light throughout the 90-minute time course, Rab11-associated vesicles are unable to move into the cytokinetic bridge and remain clustered within the cell body, inhibiting the ability of this cell to abscise (Fig. 5b). Under conditions of CRY2-mCherry and CIB1-mCerulean-Rab11 expression with 488 nm blue-light exposure, a significant increase in the percentage of binucleated cells occurred when compared with cells not exposed to the blue light (Fig. 5c). Previous in-vitro studies reported that increases in binucleate formation can be indicative of cytokinesis or abscission failure^39^. We generated Rab11-null cells (Supplementary Fig. 5c–d) and found that although cytokinesis occurred as expected, binucleate formation occurred after the formation of the cytokinetic bridge due to abscission failure (Supplementary Fig. 5d–f and Supplementary Movie 8). Rab11-null cells had a significantly higher percentage of binucleate cells when compared with control (Supplementary Fig. 5d–e), similar to clustering Rab11 in vitro using optogenetics (Fig. 5a–c).

We next sought to determine whether this binucleate phenotype could be recapitulated in zebrafish. We injected mRNA into zebrafish embryos to express CRY2-fluorescent protein (FP, mCherry, or no FP) and CIB1-FP-Rab11 (FP, either mCerulean or mCherry; Supplementary Fig. 5g). Uninjected embryos (control), embryos injected with CRY2-FP mRNA only (control), CIB1-FP-Rab11 mRNA only (control), or injected with both CRY2-FP and CIB1-FP-Rab11 mRNA (experimental) were exposed to normal light or 488 nm blue-light conditions starting at 50–60% epiboly until a late lumen expansion stage (14 hpf, experimental protocol diagram in Supplementary Fig. 5g). Embryonic lethality during optogenetic experiments was similar in all injection groups (Supplementary Fig. 5h), suggesting that acute clustering of Rab11 membranes did not result in embryo mortality. Embryos were fixed and the number of binucleate cells were evaluated (Fig. 5d–f). Strikingly we found a significant increase in the number of binucleated cells in KV under experimental conditions (Fig. 5f), suggesting that clustering Rab11 vesicles in vivo blocks abscission.

### Rab11 vesicles are required for lumen formation

**Fig. 6:**
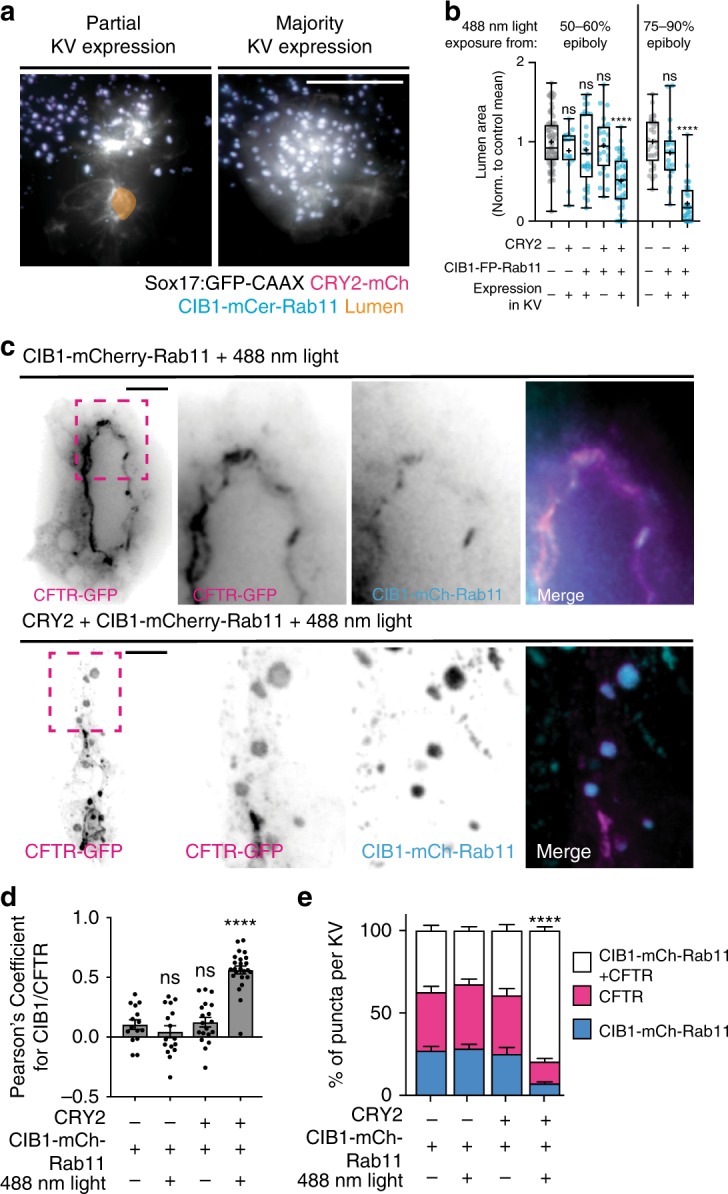
Optogenetic clustering of Rab11 during KV development results in abnormal lumen formation and perturbed polarity establishment. **a** Representative 3D renderings of KV under conditions of CRY2-mCherry/CIB1-mCerulean-Rab11 plus 488 nm light with partial (top) or majority KV mRNA expression (bottom). 3D rendering with lumen trace (orange), cell membrane (GFP-CAAX, white), CRY2-mCherry (magenta), and CIB1-mCerulean-Rab11 (cyan) shown. Bar, 50 μm. **b** Box and whisker plot depicting two-dimensional lumen area normalized to uninjected control values plus or minus 488 nm light beginning at 50–60% epiboly (left, *n* > 15 embryos) or 75–90% epiboly (right, *n* > 21 embryos). Dots represent individual KV values. Whiskers denote minimum and maximum values, 25th and 75th percentiles denoted by box boundaries. Median denoted by line within box and mean denoted by plus sign. One-way ANOVA with Dunnett’s multiple comparison test, compared with uninjected embryos. Statistical results detailed in Supplementary Table 5. **c** Representative 3D renderings of KV in CFTR-GFP (magenta) embryos under conditions of CIB1-mCherry-Rab11 (cyan, top) or CRY2 + CIB1-mCherry-Rab11 (cyan) + 488 nm light exposure (bottom). Dashed box represents insets shown at the right. Bars, 20 μm. **d** Bar graph depicting the Pearson’s coefficient for CFTR-GFP and CIB1-mCherry-Rab11 in embryos treated plus or minus 488 nm light exposure. ANOVA with Dunnett’s multiple comparison test, compared with embryos expressing CIB1-mCherry-Rab11 minus 488 nm exposure. Statistical results detailed in Supplementary Table 5. Mean displayed ± SEM. Dots represent individual values. **e** Bar graph depicting the percentage of puncta per KV expressing CFTR-GFP (magenta), CIB1-mCherry-Rab11 (cyan), or both (white). One-way ANOVA with Dunnett’s multiple comparison test completed for each cluster type, compared with percentages from embryos expressing CIB1-mCherry-Rab11 under normal light conditions. Statistical results detailed in Supplementary Table 5. **d**, **e** *n* > 10 embryos analyzed from five experiments. Mean displayed ± SEM. Dots represent individual values.

We next examined whether the clustering of Rab11 membranes resulted in KV lumen formation defects. Due to the mosaic nature of mRNA expression in zebrafish, embryos were categorized into five groups: uninjected (control), CRY2-FP mRNA only (control), CIB1-FP-Rab11 mRNA only (control), CRY2-FP plus CIB1-FP-Rab11 mRNA without KV expression (control), and CRY2-FP plus CIB1-FP-Rab11 mRNA with KV expression (experimental). Injected embryos were exposed to 488 nm light at either 50–60% epiboly or 75–90% epiboly (Fig. 6a, b and Supplementary Fig. 5g). Zebrafish developmental speed can vary due to variations in ambient room temperature^40^. To control for this, lumen area was normalized to the mean of uninjected control embryos within each clutch. This minimized the variation in lumen area due to differences in clutch developmental speed, as control groups demonstrated a range in basal lumen area dependent on the clutch (Supplementary Fig. 6b).

In double-injected embryos where KV cells have clustered Rab11-associated membranes (488 nm exposure beginning at 50–60% or 75–90% epiboly), significant defects in KV lumen formation occurred such as decreased lumen area or an inability to form a lumen at all (Fig. 6a, b and Supplementary Fig. 6b) compared with control conditions (Fig. 6b and Supplementary Fig. 6a–b). When clustered Rab11 membranes only occurred in a proportion of KV cells, lumen formation in the non-clustered areas occurred (Fig. 6a, left). In embryos with clustered Rab11 in cells surrounding KV, but not KV cells, KV lumen size was comparable to unclustered-Rab11 control conditions (Fig. 6b). Overall, these findings suggest that acute inhibition of Rab11-associated vesicles within KV-destined cells disrupts lumen formation.

Rab11 is involved in the targeted apical exocytosis of cystic fibrosis transmembrane conductance regulator (CFTR) to the apical membrane in mammalian tissue culture^41^. In zebrafish, CFTR apical localization is required for KV lumen expansion^42^. When monitoring the positioning of cytokinetic bridge/midbody in relation to CFTR in KV, we found that CFTR organizes on either side of the cytokinetic bridge midbody during MET (Supplementary Fig. 6c). CFTR-GFP was highly dynamic within regions proximal to the midbody, where a significant increase in CFTR-GFP integrated intensity was measured over time adjacent to the midbody (Supplementary Fig. 6d–e). These findings suggest a model that cytokinetic bridges provide a locale for directed membrane transport of apical polarity proteins (e.g., CFTR) for lumen establishment.

To test whether CFTR utilizes Rab11 for its apical distribution in KV, we examined whether Rab11-associated vesicles trapped CFTR when optogenetically clustered during KV formation. CIB1-FP-Rab11 mRNA-injected embryos were compared with embryos injected with CRY2 plus CIB1-FP-Rab11 mRNA. Both groups of embryos were exposed to 488 nm light during late epiboly until a fully developed KV should be formed (described in Supplementary Fig. 5g). Under conditions where only CIB1-FP-Rab11 mRNA was injected, CFTR-GFP clearly organizes to the apical membrane surrounding the lumen and a population of it colocalizes with CIB1-FP-Rab11 (Fig. 6c, top). However, under conditions of optogenetic clustering of Rab11, CFTR-GFP is trapped in Rab11 membrane-associated clusters and is unable to organize at the the apical membrane (Fig. 6c, bottom). Under these conditions, there is a significant increase in CFTR-GFP colocalization with CIB1-FP-Rab11 compared with non-clustered controls (Fig. 6d). Under control conditions (CIB1-FP-Rab11 plus or minus 488 nm light, CRY2 + CIB1-FP-Rab11 minus 488 nm light), we found that the percentage of puncta per KV that contained both CIB1-FP-Rab11 and CFTR was <40%. However, under experimental conditions of CRY2 plus CIB1-FP-Rab11 plus 488 nm light exposure, we found a significant increase in CIB1-FP-Rab11 puncta that contained CFTR (79.4 ± 9.87%; Fig. 6e). These findings suggest that CFTR-GFP utilizes Rab11-associated vesicles for its delivery to the apical membrane during KV formation. This is likely occurring both during abscission and in cells post abscission. It also presents an interesting model, where premature severing of the cytokinetic bridge (Fig. 4) limits the time for CFTR trafficking to the cytokinetic bridge to create an apical membrane. CFTR is a master regulator of fluid secretion through control of chloride transport to generate osmotic gradients that drive the movement of water through a tissue^43^. Here we propose that when abscission occurs prematurely or Rab11-associated vesicles carrying CFTR are clustered, CFTR cannot assemble at the apical membrane resulting in a loss of fluid flow and defects in lumen formation and/or expansion.

### CFTR transport to cytokinetic bridge aids in lumenogenesis

**Fig. 7:**
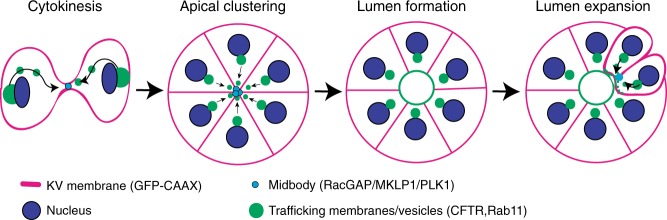
Targeted membrane transport of CFTR towards the cytokinetic bridge is used to establish a lumen. Model depicting lumen formation through Rab11-mediated vesicle transport to the cytokinetic bridge. KV membrane (GFP-CAAX—magenta), midbodies (RacGAP/MKLP1/PLK1—cyan), vesicles (CFTR/Rab11—green), and nuclei (blue) are shown.

In conclusion, these studies have highlighted the importance of cell division during the development of KV and the de novo formation of its lumen. We provide evidence that cell division is upregulated in cells destined for KV and these cells retain their cytokinetic bridges post division. The cytokinetic bridges are then projected to the site of future lumen formation during rosette formation/apical clustering, where Rab11-associated vesicles can traffic important apical polarity components to the bridge during epithelialization to allow for lumen formation (Fig. 7).

## Methods

See Supplementary Tables for list of key resources.

### Fish lines

Zebrafish lines were maintained using standard procedures approved by the Syracuse University IACUC (Institutional Animal Care and Use Committee) (protocol #18-006). Embryos were staged as described in ref. ^1^. See Supplementary Table 4 for a list of transgenic zebrafish lines used.

### Plasmid and mRNA constructs

Plasmids were all made using Gibson Cloning methods (NEBuilder HiFi DNA Assembly Cloning Kit (NEB no E5520S)) and maxi-prepped before injection (BioBasics Cat: 9K-006-0023). mRNA was made using mMESSAGE mMACHINE™SP6 transcription kit (Invitrogen AM1340). See Supplementary Table 3 for a list of plasmid constructs used and concentrations injected.

### Imaging

A SP5 or SP8 (Leica, Bannockburn, IL) laser scanning confocal microscope was used throughout this manuscript. An HC PL APO ×20/0.75 IMM CORR CS2 objective, HC PL APO ×40/1.10 W CORR CS2 0.65 water-immersion objective, and an HCX Plan Apochromat ×63/1.40-0.06 NA oil objective were used. Images were acquired using LAS-X software. A Leica DMi8 (Leica, Bannockburn, IL) with a X-light v2 confocal unit spinning disk was also used, equipped with an 89 North–LDI laser and a Photometrics Prime-95B camera. Optics used were either ×10/0.32 NA air objective, HC PL APO ×63/1.40 NA oil CS2, HC PL APO ×40/1.10 NA WCS2 CORR, a ×40/1.15 NA Lamda S LWD, or ×100/1.4 NA HC Pl Apo oil-immersion objective. In addition, a Nikon Eclipse Ti-E microscope using a Hammamatsu C9100-50 EMCCD camera coupled to a PerkinElmer spinning disk confocal system was used with a CFI Apo LWD Lambda S ×20 water-immersion objective or a CFI Apo Lambda S LWD ×40 water-immersion objective. Images were acquired using Volocity software. STED imaging was performed using a Leica TCS SP8 (Leica, Bannockburn, IL) equipped with STED 3×, a supercontinuum laser (white light laser 470–670 nm) for excitation, 592/546/600 nm STED depletion lasers, and an HCS PL APO ×100/1.40 oil STED white objective. Images were acquired using the Leica LAS software and post-image processing of STED images was performed using SVI Huygens deconvolution software.

### Laser ablation

*Tg(sox17:GFP-CAAX)* zebrafish embryos were injected with 300 pg of MKLP1-mKate mRNA at the one-cell stage. Embryos were embedded in agarose at the 1-somite stage and imaged on either an Andor Dragonfly spinning disk confocal microscope with a pulsed nitrogen pumped tunable dye laser at 100%, or X-light v2 Confocal Unit spinning disk with VisiView kinetics unit coupled to a 355 nm pulsed laser used at 50% both equipped with a ×40 1.15 NA water objective. An image was obtained prior to laser ablation to record midbody positioning within the embryo. Ablation conditions included midbodies ablated within KV or outside KV, KV cytosol, or KV cell–cell interfaces. Images of KV post ablation were captured using the 488 nm and 561 nm lasers, obtained a *z*-stack with a 0.8 μm step size every 2 min.

### Zebrafish optogenetics experiments

Optogenetic experiments were performed by injecting CRY2-mCherry and/or CIB1-mCerulean-Rab11 (or CRY2 and/or CIB1-mCherry-Rab11) mRNA into zebrafish embryos at the one-cell stage. Embryos were exposed to 488 nm light using the NIGHTSEA fluorescence system from 60% or 75–90% epiboly (late exposure experiments) until six- to eight-somite stage. Embryos were either fixed with 4% paraformaldehyde (PFA) + 0.5% Triton X-100 in phosphate buffered saline (PBS) or incubated overnight in the absence of 488 nm light to evaluate death rates. Fixed embryos were then imaged on a confocal microscope as described above.

### Pharmacological treatments

For Nocodazole and BI2536 treatments, zebrafish embryos were dechorionated and soaked in the desired concentration of drug diluted in zebrafish embryo water (refer to Fig. 1 and Supplementary Fig. 1). Embryos were manually dechorionated and treated from 60% epiboly until six- to eight-somite stage on petri plates coated with 3% agarose, when they were washed with embryo water and fixed in 4% PFA containing 0.5% Triton X-100 overnight at 4 °C. Staining, imaging, and lumen size quantification were then completed as described.

### Immunofluorescence of zebrafish embryos

Zebrafish embryos were fixed using 4% PFA containing 0.5% Triton X-100 overnight at 4 °C. Zebrafish were then dechorionated and incubated in PBST (PBS + 0.1% Tween) for 30 min. Embryos were blocked using a Fish Wash Buffer (PBS + 1% bovine serum albumin (BSA) + 1% DMSO + 0.1% Triton X-100) for 30 min followed by primary antibodies incubation (antibodies diluted in Fish Wash Buffer in concentrations stated in Supplementary Table 2) either overnight at 4 °C or 3 h at room temperature. Embryos are then washed five times in Fish Wash Buffer before incubating with secondary antibodies for 3 h at room temperature. After five more washes, embryos were incubated with 4′,6-diamidino-2-phenylindole (DAPI; NucBlue® Fixed Cell ReadyProbes® Reagent) for 30 min. For imaging, embryos were either halved and mounted on slides using Prolong Diamond (Thermo Fisher Scientific catalog number P36971) or whole-mounted in 2% agarose (Thermo Fisher catalog number 16520100).

### Cell culture

The 3D MDCK cultures were grown in Dulbecco’s modified Eagle’s medium (Gibco™) supplemented with 10% Seradigm FBS (VWR) and 1% penicillin–streptomycin (10,000 U/ml) (Gibco™) with 40% Matrigel (Fisher catalog number CB40234C; Corning number 356237).

### Rab11 optogenetic clustering in HeLa cells

HeLa cells were transfected with CIB1-mCerulean-Rab11 and CRY2-mCherry using Mirus TransIT-LT1 and then synched at prometaphase in nocodazole (100 nM) and released after 6 h in the presence or absence of 488 nm light. Cells were imaged on a spinning disk confocal microscope. Images of dividing cells were acquired for a time-lapse series or cells were imaged 2 h post release to quantify binucleate cells.

### Rab11 CRISPR

HeLa cells expressing FIP3-GFP stably were used throughout the study, maintained at 37 °C with 5% CO_2_. Rab11A CRISPR vector (Santa Cruz SC-400617) and Rab11A HDR vector (Santa Cruz SC-400617-HD) were transfected into cells using the Mirus TransIT-LT1 transfection reagents (catalog number MIR2305) using the manufacturer’s specifications. Cells were grown in puromycin selection medium (5 μg/ml). Three single clones were isolated and tested for Rab11 levels using western blotting. HeLa cells are maintained at 37 °C with 5% CO_2_.

### Immunofluorescence of 3D acini

Using a pipette, media was carefully removed from cultures. Cultures were rinsed with PBS and fixed with 4% PFA at room temperature for 30 min with light shaking. The PFA was removed and replaced with fresh PFA for an additional 30 min with light shaking. After PFA was removed, 50 mM NH_4_Cl was added for 10 min. Cells were washed with PBS for 30 min, with light shaking, and then treated for 5 min with 0.1% Triton X, blocked with PBST (PBS, 1% BSA, 0.5% Triton X-100), and incubated with primary antibodies for 4 h at room temperature. Cultures were washed three times with PBST and incubated with secondary antibodies for 4 h at room temperature. For actin and DAPI staining, acini were incubated with ActinRed 555vReady Probes reagent (Thermo Fisher Scientific R37112) and NucBlue Fixed Cell Stain from Ready Probes (Thermo Fisher Scientific R37606) for 30 min. Cultures were kept in PBS containing DABCO (1,4-Diazabicyclo [2.2.2] octane) antifade reagent (200 μM) for imaging. See Supplementary Table 2 for a list of antibodies and concentrations used.

### Image and data analysis

Images were processed using both FIJI/ImageJ software, IMARIS (Bitplane), and/or Adobe Photoshop. Angles were calculated using FIJI/ImageJ software and Microsoft Excel. All graphs were generated and statistical analysis performed using GraphPad Prism software. The 3D images, movies, and surface rendering were performed using Bitplane IMARIS (Surface, Smoothing, Masking, and Thresholding functions).

### Surface renderings

Imaris surface renderings were created through the manual surface protocol by outlining fluorescence regions of interest (ROIs) using the Isoline function for each *z*-plane and time point. Once the surface rendering was created for each cell, individual cell renderings were pseudocolored and each frame was captured. To isolate and pseudocolor specific cells, the same surface-rendering protocol was completed and masks were created from the surface renderings to isolate the new channel.

### CFTR intensity measurements

To measure the integrated density of CFTR at the cytokinetic midbody, rectangular ROIs were drawn around the midbody (MKLP1). The larger ROI (ROI^L^) is used to measure background, whereas the center, smaller ROI (ROI^S^) measures the CFTR signal. The following equation was used: integrated intensity of ROI^S^ − ((integrated intensity of ROI^L^ − integrated intensity of ROI^S^)*(area ROI^S^/(area ROI^L^ − area ROI^S^)))^44^. Intensities were normalized to the final intensity at 4 min (percentage).

### Calculation of spindle orientation in relation to lumen

Spindle orientation was measured during the apical clustering and lumen formation stages of KV development. As shown in Supplementary Fig. 3e, a line was drawn through the DNA plate of a metaphase cell in KV (solid black line). A second line was then drawn perpendicular to the first line to denote the position of the mitotic spindle poles (dashed black line). A third line was drawn passing through the center of KV and the center of the metaphase DNA plate (dashed gray line). Lastly, the angle between the dashed gray line and the dashed black line were calculated to determine the spindle position in relation to the KV center.

### Lumen area quantifications

Prior to lumen area measurements, images were turned using Imaris software such that the equatorial plane of the lumen could be measured, resulting in a representative lumen area measurement regardless of initial embryo positioning during imaging. This dataset was then transferred to FIJI/ImageJ for lumen area calculations. A region was drawn around the lumen perimeter and area calculated using the measure function. Where applicable, values were normalized to the control mean by dividing each lumen area by the mean value of the control lumens for that particular experiment. This controlled for KV size fluctuations based on slight differences in ambient room temperature and difference growth rates of clutches in different experimental setups.

### Mitotic index and cell number calculations

Mitotic index and cell number counts were completed with embryos after a DAPI stain and/or antibody staining with a phospho-H3 antibody. For mitotic index, the number of mitotic cells was divided by the total number of cells to result in a percentage of mitotic cells out of the entire population.

### Colocalization quantification

For optogenetic experiments, colocalization quantification was performed using Imaris software. In the “Colocalization” menu, a ROI around KV was defined by masking the channel depicting CFTR fluorescence. CFTR was defined as Channel 1 and CIB1-FP-Rab11 was defined as Channel 2. Threshold values were calculated with the “Automatic Threshold” option to define colocalization parameters and the “Pearson’s coefficient in colocalized volume” was recorded for each embryo. In addition, optogenetic clusters were scored for the presence of CFTR, CIB1-FP-Rab11, or both, and this was presented as percentages per embryo.

### Statistics and reproducibility

Unpaired, two-tailed Student’s *t*-tests, Mann–Whitney, and one-way analysis of variance analyses were performed using GraphPad Prism software; ****p-value < 0.0001, ***p-value < 0.001, **p-value < 0.01, *p-value < 0.05. See Supplementary Table 5 for detailed information regarding statistics.

All graphs, micrographs, images, and blots in this study are representative of at least three independent experiments.

### Reporting summary

Further information on research design is available in the Nature Research Reporting Summary linked to this article.

## Supplementary information

Supplementary Information

Description of Additional Supplementary Files

Supplementary Movie 1

Supplementary Movie 2

Supplementary Movie 3

Supplementary Movie 4

Supplementary Movie 5

Supplementary Movie 6

Supplementary Movie 7

Supplementary Movie 8

Reporting Summary

## Data Availability

The source data underlying all graphs in this study have been provided in a Source Data file.

## Source Data

Source Data
