# Supplementary material for: Cytokinetic bridge triggers de novo lumen formation in vivo
Source: Nat Commun. 2020 Mar 9;11:1269. doi: 10.1038/s41467-020-15002-8 (PMC7062744; doi:10.1038/s41467-020-15002-8)
Supplement: Supplementary file 1 — Supplementary Information [file 41467_2020_15002_MOESM1_ESM.pdf]

Supplementary Information for

Cytokinetic bridge triggers *de novo* lumen formation *in vivo*

Rathbun LI[1], Colicino EG[1,2,6], Manikas J[1], O'Connell J[1], Krishnan N[1], Reilly NS[3], Coyne S[2,4], , Erdemci-Tandogan G[5], Garrastegui A[1], Freshour J[1], Santra P[2], Manning ML[5], Amack JD[2], Hehnly H[*1]

[1]Biology Department, Syracuse University, Syracuse NY
[2]Department of Cell and Developmental Biology, SUNY Upstate Medical School, Syracuse NY
[3]Department of Physics and Astronomy, University of Rochester, Rochester NY
[4]Department of Biology, SUNY Geneseo, Geneseo NY
[5]Department of Physics, Syracuse University, Syracuse NY
[6]Current location: Department of Cell and Developmental Biology, University of Michigan Medical School Ann Arbor, MI
[*]Corresponding author: hhehnly@syr.edu

This PDF file includes:

Supplementary Figures 1-6

Supplementary Tables 1-5

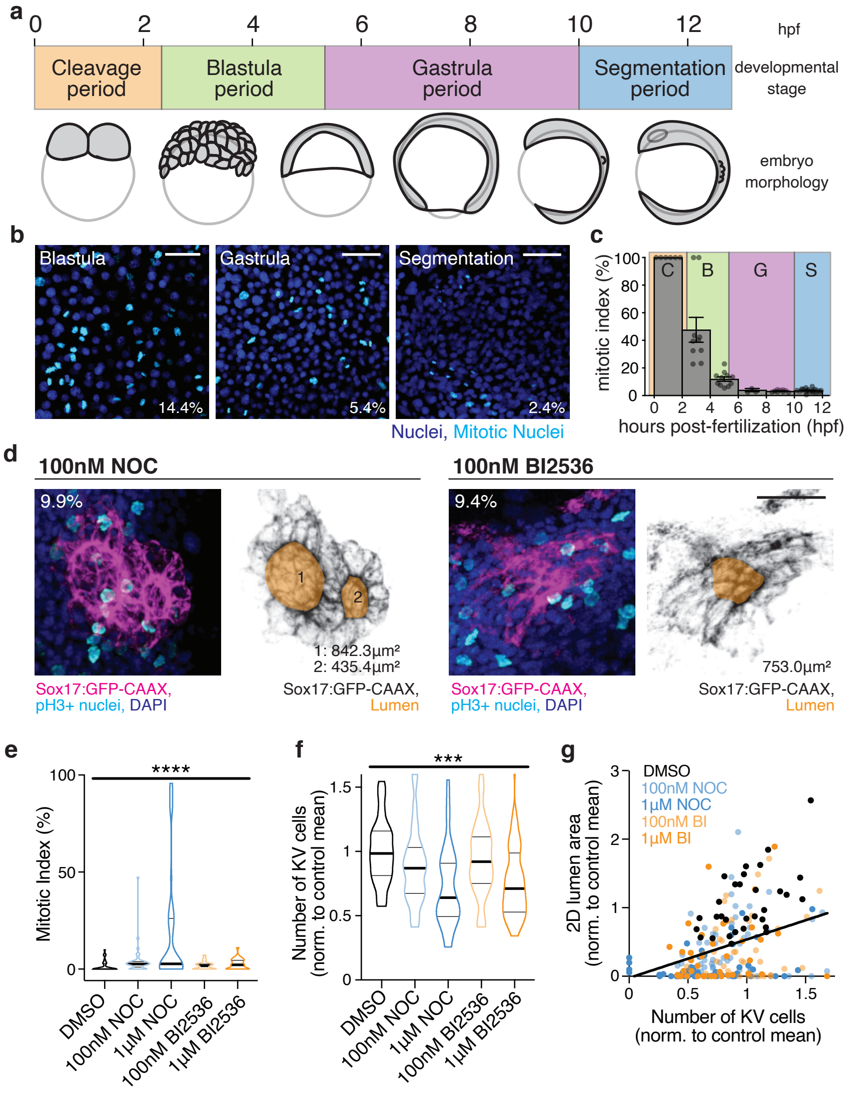

Supplementary Figure 1. Mitosis is required for lumen formation. (a) Timeline depicting zebrafish embryo development from 0-12 hours post-fertilization (hpf, top) and models with embryo morphology during these stages (bottom). Adapted from 19. **(b)** Representative confocal projection of nuclei during the blastula, gastrula, and segmentation periods. Mitotic nuclei (cyan) and non-dividing nuclei (blue) displayed. Percentages indicate mitotic index of image. Bar, 20µm. **(c)** Histogram displaying mitotic index from 0-12hpf. Cleavage (C, orange), blastula (B, green), gastrula (G, purple), and segmentation (S, blue) periods highlighted. n > 80 embryos ± SEM. N=6 (0-2hpf), 10 (2-4hpf), 11 (4-6hpf), 3 (6-8hpf), 9 (8-10hpf), 32 embryos (10-12hpf) from 11 clutches. **(d)** Representative 3D renderings of KV under conditions of microtubule inhibition (100nM nocodazole) or PLK1 inhibition (100nM BI2536). Sox17:GFP-CAAX (magenta), pH3-positive nuclei (cyan), and DAPI (blue) shown on left. Sox17:GFP-CAAX (gray) and lumen trace (orange) shown on right. Bar, 20µm. Percentages indicate mitotic index of image. **(e-f)** Violin plot depicting KV mitotic index (e) or cell number (f) under conditions of DMSO (black), BI2536 (orange), or nocodazole (blue) treatment. Endpoints depict minimum and maximum values, quartiles depicted by thin black lines, median depicted by thick black line. (e-f) One-way ANOVA, $p<0.00001$ (****). (e) DMSO: n=27 embryos, 100nM BI2536: n=35 embryos, 1µM BI2536: n=30 embryos, 100nM nocodazole: n=39 embryos, 1 µM nocodazole: n=40 embryos per treatment over four independent experiments. $F(4,166)=8.733$, df=166. (f) DMSO: n=32 embryos, 100nM BI2536: n=40 embryos, 1µM BI2536: n=37 embryos, 100nM nocodazole: n=48 embryos, 1 µM nocodazole: n=45 embryos per treatment over four independent experiments. $F(4,197)=6.137$, df=197. **(g)** Scatter plot depicting relationship between number cells in KV (normalized to control mean, x-axis) and 2D lumen area (normalized to control mean, y-axis). DMSO (black), BI2536 (orange), and nocodazole (blue) treatment conditions included. n>31 embryos per treatment across five independent experiments. Pearson's correlation: $r=0.3653$, $p<0.0001$ (****), n=206 pairs of x,y values.

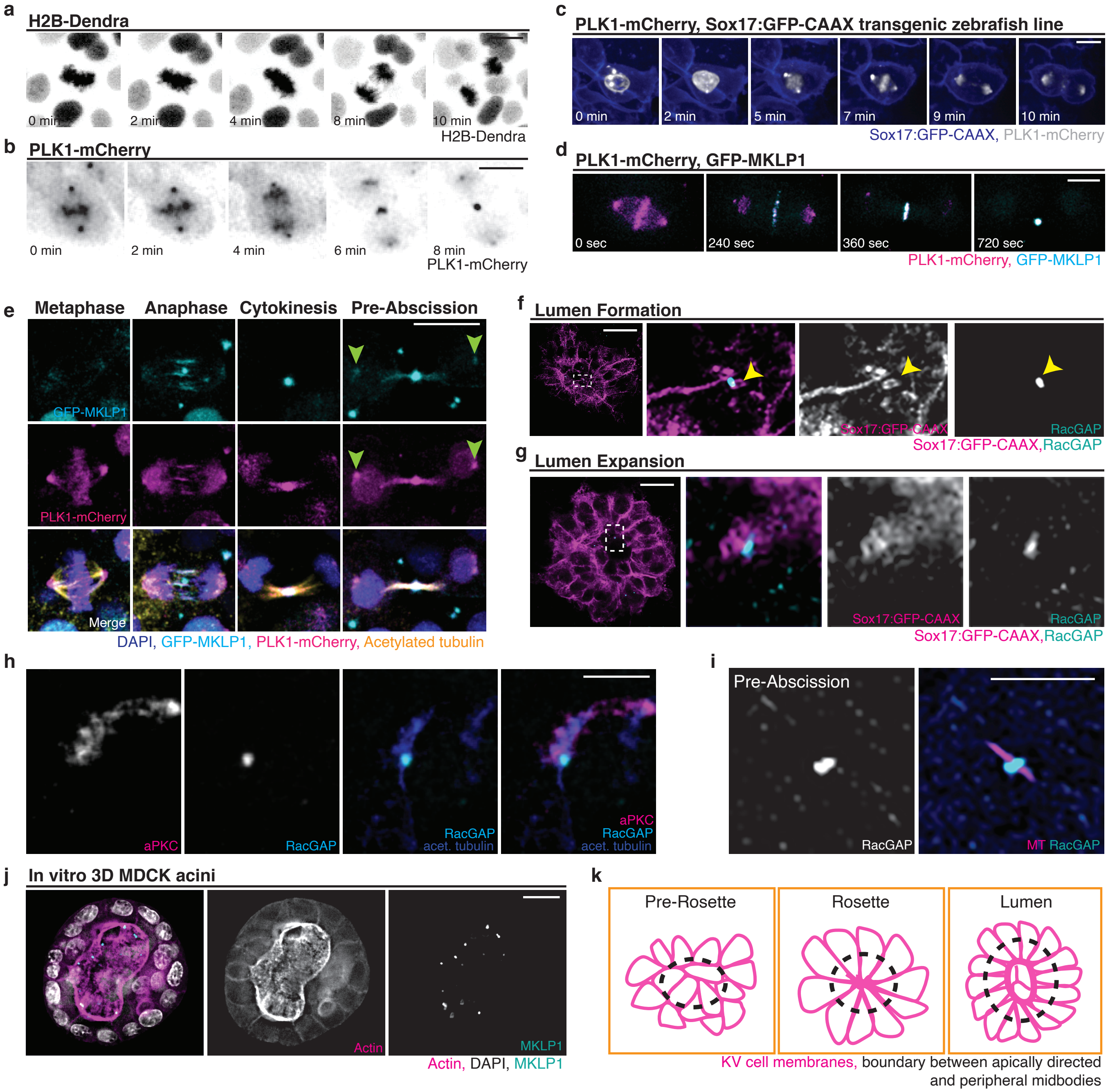

Supplementary Figure 2. Cytokinetic midbodies localize to apical membranes of lumens *in vitro* and *in vivo*. **(a-b)** Maximum confocal projections of live cell divisions shown by nuclear marker (H2B-Dendra, a) and mitotic marker (mCherry-PLK1, b). Bars, 10µm. **(c)** 3D rendering of a live Sox17:GFP-CAAX-positive cell (blue) expressing PLK1-mCherry (white) as it progresses through mitosis. Bar, 10 µm. **(d)** Maximum confocal projections of a live mitotic cell as it progresses from metaphase (0 sec) to cytokinesis (720 sec). PLK1-mCherry (magenta) and GFP-MKLP1 (cyan) depicted. Bar, 5µm. **(e)** Maximum confocal projections of fixed mitotic cells during metaphase, anaphase, cytokinesis, and pre-abscission. Nuclei (blue), MKLP1 (cyan), PLK1 (magenta), and acetylated tubulin (gold) shown. Green arrowheads depict locations of centrosomes during cytokinesis. Bar, 5µm. **(f-g)** Maximum confocal projections of fixed embryos during lumen formation and lumen expansion stages of KV development. Midbodies (RacGAP, cyan) and KV membrane (CAAX, magenta) shown. Bar, 20µm. **(h)** Maximum STED microscopy projections of fixed embryos depicting the cytokinetic midbody (RacGAP, cyan), apical polarity (aPKC, magenta), and acetylated tubulin (blue). Bar, 5µm. **(i)** Maximum projection depicting a midbody (RacGAP, cyan) and associated cytokinetic bridge (tubulin, magenta). Bar, 10µm. **(j)** MDCK 3D-acini immunolabeled for actin (magenta), midbodies (MKLP1, cyan), and nuclei (DAPI, white). Bar, 20µm. **(k)** Diagram depicting the boundary between apical midbodies (inner KV) and peripheral midbodies (outer KV) shown by dashed black line. KV cell membranes (magenta) shown at pre-rosette (left), rosette (center), and lumen (right) shown.

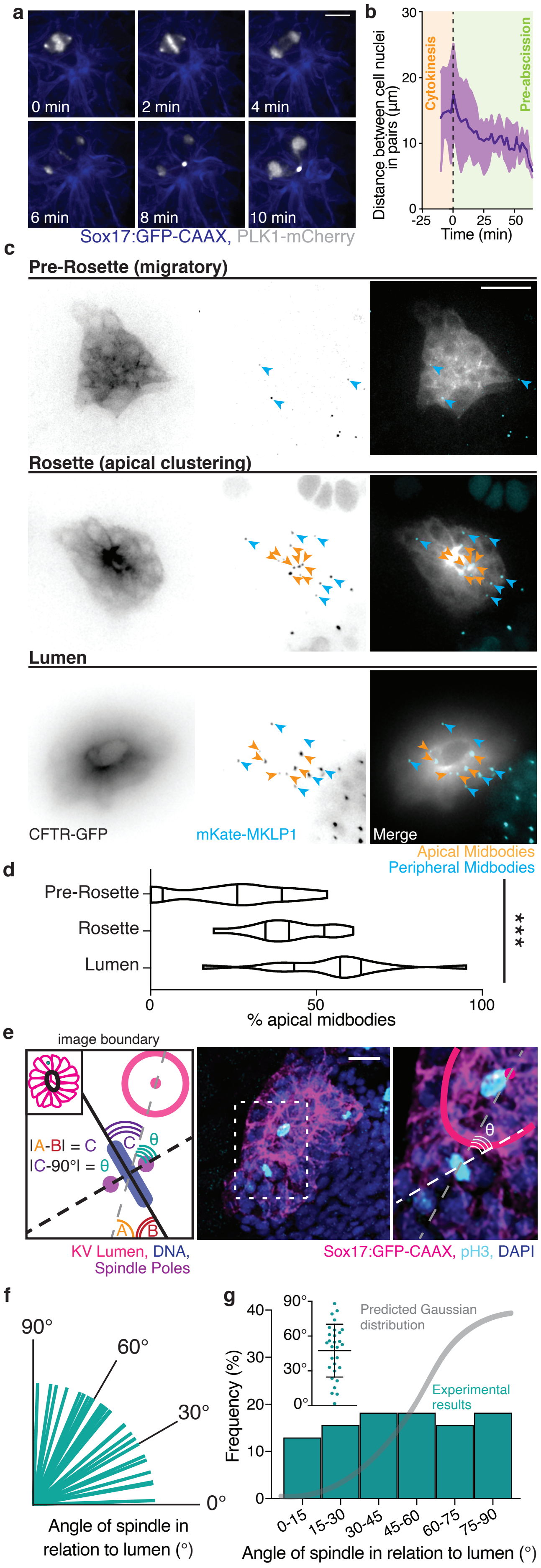

Supplementary Figure 3. Cytokinetic bridges are placed at the site of future KV lumen formation. (a) 3D renderings of a mitotic cell (PLK1-mCherry, cyan) within KV (Sox17:GFP-CAAX, magenta) during metaphase (top) and cytokinesis (bottom). Orange asterisk denotes position of forming lumen during cytokinesis. Bar, 10µm. **(b)** Distance between cell pairs quantified from cytokinesis (-10 to 0 min) through pre-abscission (0-70 min, n = 17 pairs from n = 7 embryos, range with mean shown). **(c)** Representative images of midbody localization (mKate-MKLP1, cyan) within KV (CFTR-GFP, grayscale). Pre-rosette (top), rosette (middle), and lumen (bottom) stages of KV development depicted. Orange arrowheads denote apical midbodies, cyan arrowheads denote peripheral midbodies. Bar, 20µm. **(d)** Violin plot depicting percentage of apical midbodies in live KVs at pre-rosette (n=12 embryos), rosette (n=15 embryos), and lumen (n=15 embryos) stages. Endpoints depict minimum and maximum values, quartiles depicted by thin black lines, median depicted by thick black line. n>4 independent experiments. One-way ANOVA, $p<0.0001$ (****), $F(2,39)=12.74$, df=39. **(e)** Diagram for spindle orientation calculations (left). Angle calculation example within KV (right). Bar, 20 µm. **(f-g)** Individual spindle angles (f) and angle frequency (g, n = 28 cells, n = 25 embryos) for mitotic cells during KV development. Inset, scatterplot of individual angles ± SD.

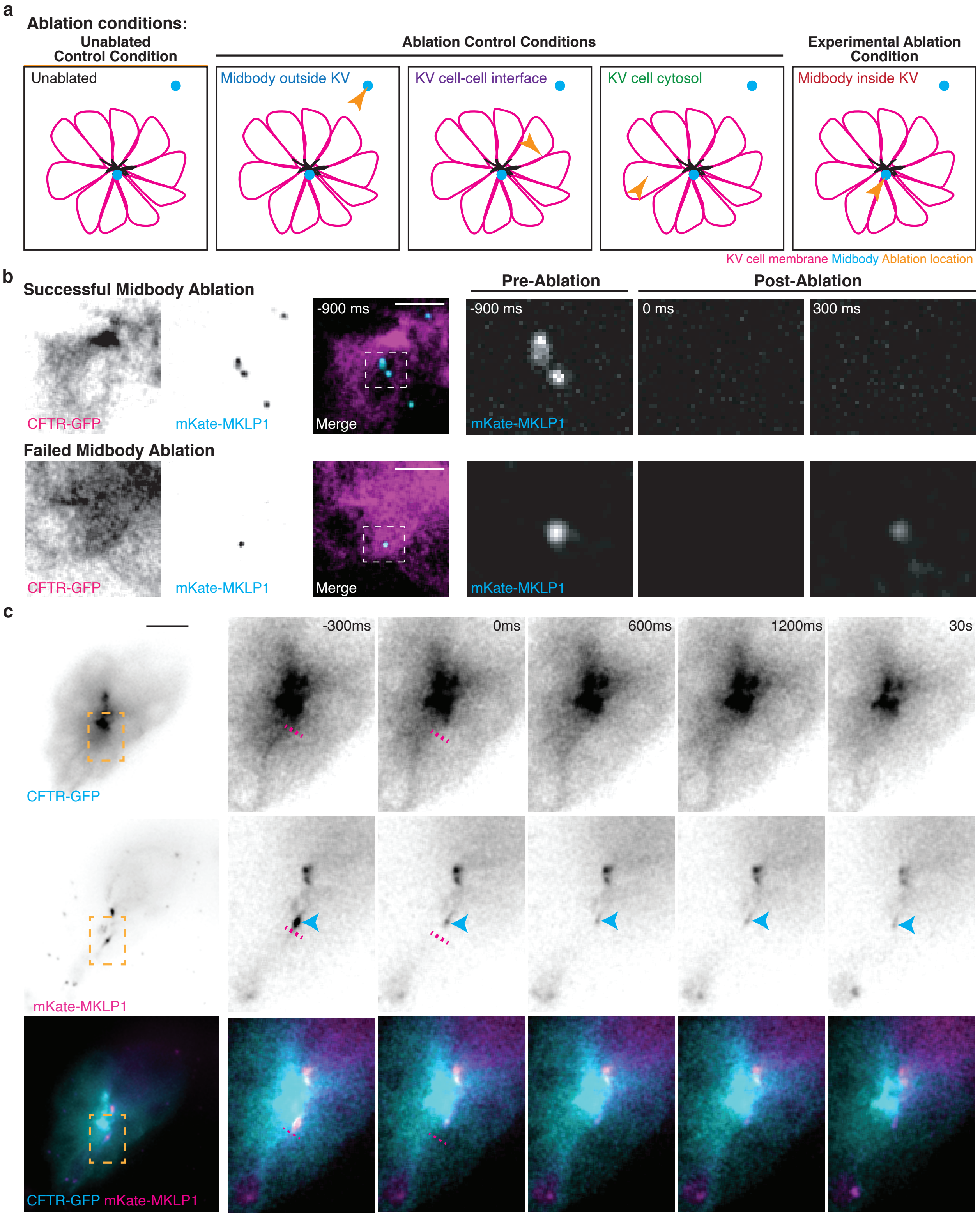

Supplementary Figure 4. Premature cleavage of the cytokinetic bridge via laser ablation results in disrupted lumen formation. (a) Diagram depicting locations of laser ablation for unablated embryos and embryos with ablation of midbody outside KV (blue), KV cell-cell interface (purple), KV cell cytosol (green), or midbody inside KV (red). KV cell membrane (magenta), midbody marker (cyan), and ablation location (orange arrowhead) shown. **(b)** Maximum projections depicting a successful (top) and failed (bottom) midbody ablation. KV marker (CFTR-GFP, magenta) and midbodies (mKate-MKLP1, cyan) shown. Pre-ablation and post-ablation images shown on right of panels (mKate-MKLP1, grayscale). Bar, 10µm. **(c)** Time lapse images depicting a single cytokinetic bridge (mKate-MKLP1 magenta) within KV (CFTR-GFP, cyan). Inset shows cytokinetic bridge magnified at 3x. Cyan arrowhead depicts midbody with ablation location shown with magenta dashed line. Bar, 20µm.

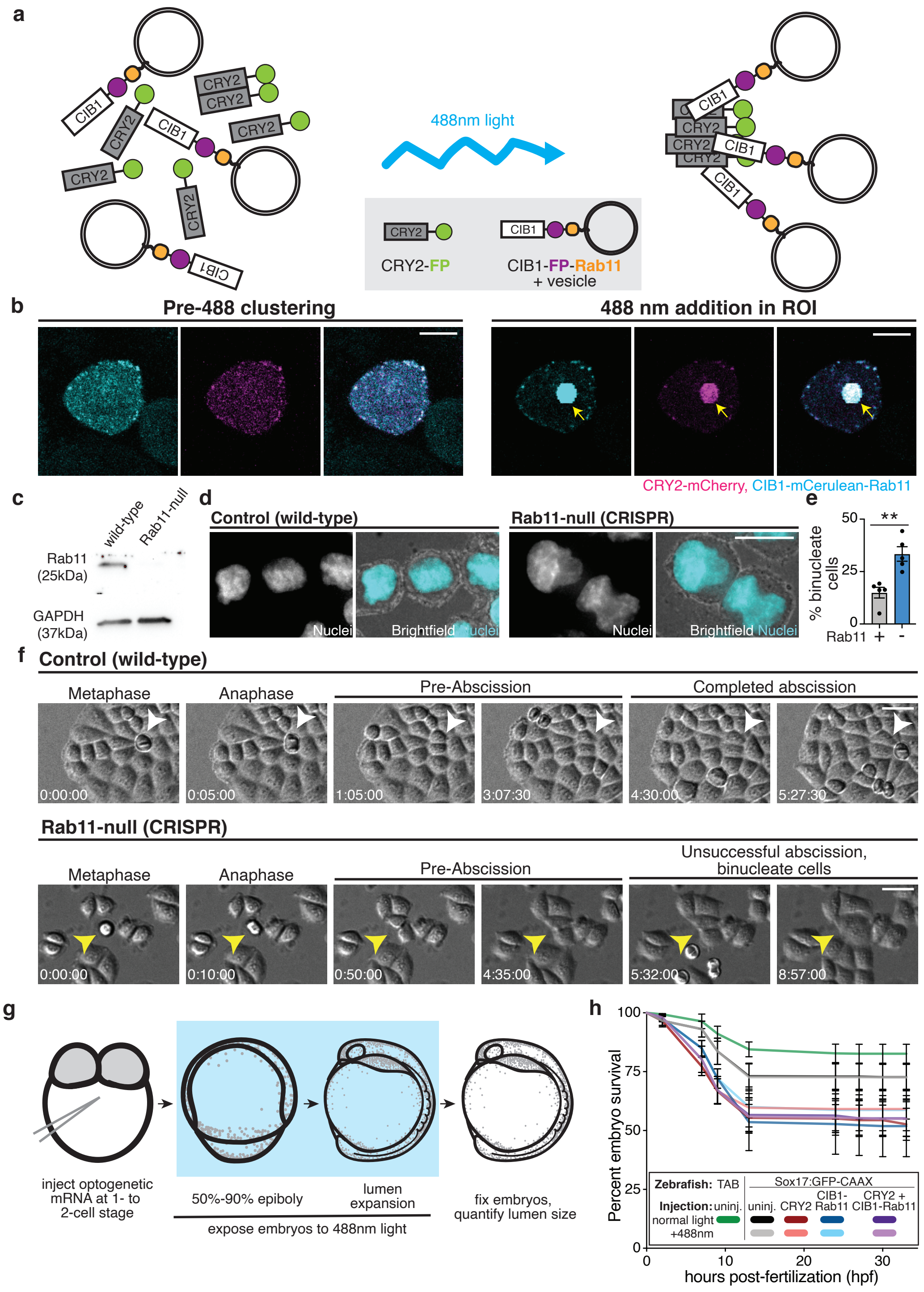

Supplementary Figure 5. Optogenetic clustering of Rab11-associated vesicles results in failed abscission *in vitro* and *in vivo*.
(a) Model depicting CRY2/CIB1 optogenetic system utilized in these studies. Since multiple fluorescent molecules are used, FP (fluorescent protein) is used in model for simplicity. CRY2-FP and CIB1-FP-Rab11 shown under conditions of no light (no clustering) or 488nm light (clustering). **(b)** Example micrograph of HeLa cells before (left) and after (right) exposure to 488nm light within a circular ROI. CRY2-mCherry (magenta) and CIB1-mCerulean-Rab11 (cyan) shown. Bar, 5µm. **(c)** Western blot depicting Rab11 protein expression in wild-type (left) and Rab11-null CRISPR HeLa cells (right). Rab11 (25kDa) and GAPDH (37kDa) loading control shown. **(d)** Representative maximum projections of nuclei in wild-type (left) and Rab11-null CRISPR cells (right). Brightfield (grayscale) and nuclei (DAPI, cyan) shown. Bar, 10µm. **(e)** Bar graph depicting percentage of binucleate cells in wild-type (Rab11-positive, gray) and Rab11-null CRISPR cells (Rab11-negative, cyan). Two-tailed Mann-Whitney p=0.0079 (**), u=0. n=100 cells across n=5 experiments. **(f)** Time lapse images of cell divisions in wild-type (control, top) and Rab11-null CRISPR cells (bottom). Metaphase, anaphase, pre-abscission stages shown with either subsequent completed (white arrowhead) or unsuccessful abscission with binucleate cell resulting (yellow arrowhead). Bar, 50µm. **(g)** Model depicting optogenetic experiment protocol. **(h)** Line graph depicting embryo survival during the first 33hpf. Wild-type (TAB) embryos (green, n=655 embryos from 3 clutches), uninjected transgenic embryos +/- 488nm light exposure (grayscale, n=831 embryos from 4 clutches), CRY2-FP-injected transgenic embryos +/- 488nm light exposure (red), CIB1-FP-Rab11-injected transgenic embryos +/- 488nm light exposure (blue), and CRY2-FP + CIB1-FP-Rab11-injected transgenic embryos +/- 488nm light exposure (purple, n=555 embryos from 4 clutches) shown. Mean ± SEM shown.

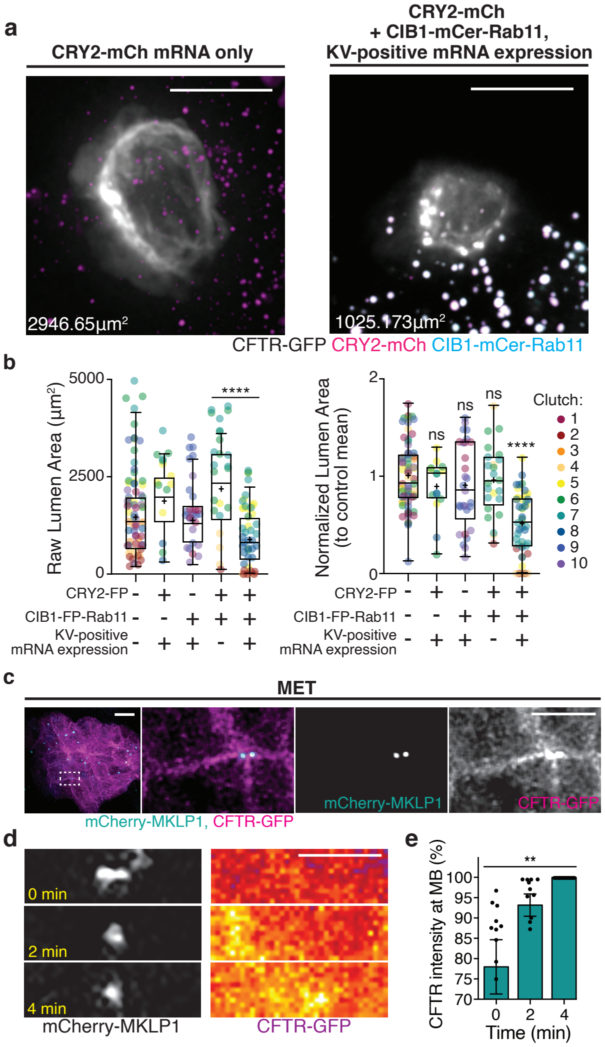

Supplementary Figure 6. Optogenetic clustering of Rab11 during KV development results in abnormal lumen formation and perturbed polarity establishment. (a) Representative 3D renderings of KV under conditions of CRY2-mCherry mRNA only, or CRY2-mCherry + CIB1-mCerulean-Rab11 with KV-positive mRNA expression. KV cells (CFTR-GFP, white), CRY2-mCherry (magenta), and CIB1-mCerulean-Rab11 (cyan) shown. Bars, 50 µm. **(b)** Box and whisker plots comparing raw (left) and normalized (right) lumen area resulting from optogenetic experiments (Figure 6b). Normalized data shown as a ratio compared to uninjected control mean. Whiskers denote minimum and maximum values, 25th and 75th percentiles denoted by box boundaries. Median denoted by line within box, mean denoted by plus sign. Data from 10 individual clutches shown by colors described in legend. n>15 embryos per treatment. Results from unpaired, two-tailed Student's t-test of raw data set (left) and one-way ANOVA with Dunnett's multiple comparison test in normalized data described in detail in Supplementary Table 5. **(c)** Midbodies (mCherry-MKLP1, cyan) localize at sites of apical polarity (CFTR-GFP, magenta) during MET stage of KV development. Bar, 20 µm (top), 10 µm (bottom). **(d)** Apical polarity (monitored by CFTR, Fire LUT) increases within a 4 minute time frame adjacent to the cytokinetic midbody (mCherry-MKLP1). Bar, 5 µm. **(e)** Quantification of CFTR-GFP intensity over time. n = 12 midbodies ± SEM, n = 4 embryos, One-way ANOVA, $p = 0.0023$ (**), $F(2,33) = 7.323$, df=33.

Supplementary Tables

Supplementary Table 1: Key Resources Table

Reagent Type (species or resource)	Designation	Source
General Genes Examined (zebrafish)	Mitotic kinesin-like protein (MKLP1), Histone H2B (H2B)	Gene ID #: MKLP1- 30627<br>H2B- 100334869, 100334559, 100329560, 100329290
Organ of Asymmetry localized genes	cystic fibrosis transmembrane conductance regulator (CFTR)	Gene ID #:<br>CFTR- 559080
Commercial Assay/Kit	NEBuilder HiFi DNA Assembly Cloning Kit (NEB no E5520S), mMESSAGE mMACHINE™SP6 transcription kit (Invitrogen AM1340)	-https://www.neb.com/products/e5520-nebuilder-hifi-dna-assembly-cloning-kit#Product%20Information<br>-https://www.thermofisher.com/order/catalog/product/AM1340
Software/Algorithm	ImageJ/FIJI, Bitplane IMARIS	Bitplane
Drugs	BI2536, nocodazole	BI2536 (Selleck Chemicals S1109), nocodazole (ACROS Organics 358240500)

Supplementary Table 2: Antibodies

Name	Dilution	Company/Cat. No
Anti-GFP	1:200	Abcam
phospho-Histone 3 (ser10)	1:200	Cell Signaling (9701S)
MKLP1 (N-19)	1:200	Santa Cruz (sc-867)
aPKC (zeta)	1:200	Santa Cruz (sc-216)
RacGAP	1:200	Abcam (ab2270)
α-tubulin conjugated with FITC	1:200	Sigma Aldrich (F2168)
PLK1	1:200	Cell Signaling Technology (4513S)
Acetylated tubulin	1:200	Sigma-Aldrich (45-T6793-100UL)
ZO-1	1:200	Invitrogen (484333A)
Rab11	1:500	Cell Signaling Technology (3539S)
GAPDH-HRP	1:40000	Sigma-Aldrich (45-G9295-25UL)

Supplementary Table 3: Plasmid Constructs

Construct	Backbone	Tag	Injection Type	Concentration [pg]
H2B	pCS2	Dendra	mRNA	95
MKLP1	pCS2	GFP/mKate/ mCherry	Plasmid	15
MKLP1	pCS2	mKate/ mCherry	mRNA	300
CRY2	pCS2	mCherry/untagged	mRNA	100
CIB1-Rab11	pCS2	mCerulean/mCherry	mRNA	100
PLK1	pCS2	mCherry	mRNA	100

Supplementary Table 4: Zebrafish Transgenic Lines

Type	Name	Source
Wild-Type	TAB	Zebrafish International Resource Center (ZIRC)
Transgenic	*Tg(sox17:GFP-CAAX)^{sny101}*	Dasgupta et al., 2018
Transgenic	*Tg(sox17:GFP)*	Sakaguchi et al., 2006
Transgenic	*Tg(sox17:dsRED)*	Chung et al., 2008
Transgenic	*TgBAC(cftr-GFP)*	Navis et al., 2013

Supplementary Table 5. Detailed statistical analysis results

Figure	Category	n	Statistical test	Parameters	Result	p-value
1c	migratory	10	Unpaired Student's t-test (two-tailed)	t=5.444, df=18	****	<0.0001
	MET	9		t=2.930, df=16	**	0.0098
	AC	8		t=3.766, df=14	**	0.002
	LF	9		t=3.992, df=16	**	0.001
	LE	7		t=3.345, df=12	**	0.0058
1e	DMSO	41	One-way ANOVA with Dunnett's multiple comparison	df=241 F(4,241)=25.86	control	n/a
	100nM NOC	50			****	<0.0001
	1uM NOC	43			****	<0.0001
	100nM BI	58			****	<0.0001
	1uM BI	54			****	<0.0001
Supp1e	DMSO	27	One-way ANOVA with Dunnett's multiple comparison	df=166 F (4, 166) = 8.733	****	<0.0001
	100nM NOC	35				
	1uM NOC	30				
	100nM BI	39				
	1uM BI	40				
Supp1f	DMSO	32	One-way ANOVA with Dunnett's multiple comparison	df=197 F (4, 197) = 6.137	***	0.0001
	100nM NOC	40				
	1uM NOC	37				
	100nM BI	48				
	1uM BI	45				
Supp1g	lumen area vs. cell number	206	Pearson's correlation	r=0.3653	****	<0.0001
2e	pre-rosette	21	One-Way ANOVA	df=69, F (2, 69) = 104.7	****	<0.0001
	rosette	16				
	lumen	35				
Supp3d	pre-rosette	12	One-Way ANOVA	df=39, F (2, 39) = 12.74	****	<0.0001
	rosette	15				
	lumen	15				
4f	MB outside KV	7	One-way ANOVA with Dunnett's multiple comparison	df=25 F(3,25)=4.352	control	n/a
	KV cell cytosol	9			ns	0.7495
	KV cell-cell interface	6			ns	0.9955
	MB inside KV	7			**	0.0097
5c	CRY2 + CIB1-mCherry-Rab11	6	Mann-Whitney test (two-tailed)	U = 0	**	0.0043
	CRY2 + CIB1-mCherry-Rab11 + 488nm light	5				
5f	uninjected	5	One-way ANOVA with Dunnett's multiple comparison	df=32 F(5,32)=41.51	control	control
	uninjected + 488nm light	6			ns	0.9982
	CIB1-mCh-Rab11	5			ns	0.9795
	CIB1-mCh-Rab11 + 488nm light	9			ns	>0.9999
	CRY2 + CIB1-mCh-Rab11	7			ns	0.9628
	CRY2 + CIB1-mCh-Rab11 + 488nm light	6			****	<0.0001
Supp5e	Control vs Rab11 CRISPR	5	Mann-Whitney test (two-tailed)	U = 0	**	0.0079
6b/Supp6b early	uninjected	73	One-way ANOVA with Dunnett's multiple comparison	df=183 F(4,183)=15.43	control	n/a
	CRY2 only	15			ns	0.8467
	CIB1 only	29			ns	0.7293
	CRY2+CIB1, no KV	24			ns	0.9946
	CRY2+CIB1, +KV	47			****	<0.0001
late	uninjected	11	One-way ANOVA with Dunnett's multiple comparison	df=83 F(2,83)=58.28	control	n/a
	CIB1 only	8			ns	0.6908
	CRY2+CIB1, +KV	18			****	<0.0001
Supp6b (non-normalized data)	uninjected	73	Unpaired Student's t-test (two-tailed)	t=6.315, df=69	n/a	n/a
	CRY2 only	15			n/a	n/a
	CIB1 only	29			n/a	n/a
	CRY2+CIB1, no KV	24			****	<0.0001
	CRY2+CIB1, +KV	47				
6d	CIB1-mCh-Rab11	15	One-way ANOVA with Dunnett's multiple comparison	df=70 F(3,70)=39.97	control	control
	CIB1-mCh-Rab11 + 488nm light	16			ns	0.6562
	CRY2 + CIB1-mCh-Rab11	19			ns	0.975
	CRY2 + CIB1-mCh-Rab11 + 488nm light	24			****	<0.0001
6e (CFTR-positive)	CIB1-mCh-Rab11	10	One-way ANOVA with Dunnett's multiple comparison	df=54 F(3,54)=21.83	control	control
	CIB1-mCh-Rab11 + 488nm light	13			ns	0.7845
	CRY2 + CIB1-mCh-Rab11	13			ns	>0.9999
	CRY2 + CIB1-mCh-Rab11 + 488nm light	22			****	<0.0001
6e (CIB1-positive)	CIB1-mCh-Rab11	10	One-way ANOVA with Dunnett's multiple comparison	df=54 F(3,54)=23.21	control	control
	CIB1-mCh-Rab11 + 488nm light	13			ns	0.9744
	CRY2 + CIB1-mCh-Rab11	13			ns	0.8978
	CRY2 + CIB1-mCh-Rab11 + 488nm light	22			****	<0.0001
6e (CFTR + CIB1-positive)	CIB1-mCh-Rab11	10	One-way ANOVA with Dunnett's multiple comparison	df=54 F(3,54)=85.73	control	control
	CIB1-mCh-Rab11 + 488nm light	13			ns	0.5408
	CRY2 + CIB1-mCh-Rab11	13			ns	0.9366
	CRY2 + CIB1-mCh-Rab11 + 488nm light	22			****	<0.0001
