# Supplementary material for: Cytokinetic bridge triggers de novo lumen formation in vivo
Source: Nat Commun. 2020 Mar 9;11:1269. doi: 10.1038/s41467-020-15002-8 (PMC7062744; doi:10.1038/s41467-020-15002-8)
Supplement: Supplementary file 2 — Description of Additional Supplementary Files [file 41467_2020_15002_MOESM2_ESM.pdf]

Description of Additional Supplementary Files

File Name: Supplementary Movie 1
Description: **Mitotic index decreases during early embryo division.** Embryo expressing H2B-Dendra (white) during development.

File Name: Supplementary Movie 2
Description: **Multiple cell divisions occur during KV development.** Three-dimensional rendering of mitotic cells (PLK1-mCherry, multicolored) within KV (Sox17:GFP-CAAX, blue) over time. Cells surface rendered and masked to create multiple channels.

File Name: Supplementary Movie 3
Description: **PLK1 as a mitotic marker in live zebrafish embryos**. Three-dimensional rendering of a single cell through mitosis. PLK1-mCherry (white) and KV marker shown (Sox17:GFPCAAX, blue).

File Name: Supplementary Movie 4
Description: **MKLP1 and PLK1 decorate the cytokinetic midbody during live zebrafish cell divisions.** A single cell undergoes mitosis during zebrafish development. GFP-MKLP1 (cyan) and PLK1-mCherry (magenta) shown.

File Name: Supplementary Movie 5
Description: **Cytokinetic bridges of dividing KV cells project towards future site of lumen formation.** Mitotic cell (MKLP1-mKate, cyan) within KV (*Tg(sox17:GFP-CAAX)*, magenta) during apical clustering.

File Name: Supplementary Movie 6
Description: **Cytokinetic bridge is abscised and midbody is deposited into lumen during KV lumenogenesis.** *Tg(sox17:GFP-CAAX)* (magenta, left) zebrafish embryo expressing MKLP1-mKate (cyan, center) from apical clustering to lumen expansion stages.

File Name: Supplementary Movie 7
Description: **Premature ablation of cytokinetic bridge/midbody results in failed lumen formation.** *Tg(sox17:GFP-CAAX)* embryo (magenta) expressing MKLP1-mKate (cyan). In control embryos (top panels), lumen formation occurs approximately 10 minutes after apical clustering stage (labeled time point 0 min). When laser ablation applied to a centrally located KV midbody (bottom panels) at the apical clustering stage (0min), lumen formation fails to occur for over 200 minutes.

File Name: Supplementary Movie 8
Description: **Rab11-null CRISPR cells display binucleate phenotype resulting from unsuccessful abscission.** Live brightfield timelapse microscopy of wild-type (left) and Rab11-null CRISPR cells (right). Black arrowhead denotes successful division of wild-type cell, yellow arrowhead denotes unsuccessful abscission and the formation of a binucleate Rab11-null cell.
