# Supplementary material for: Cytokinetic bridge triggers de novo lumen formation in vivo
Source: Nat Commun. 2020 Mar 9;11:1269. doi: 10.1038/s41467-020-15002-8 (PMC7062744; doi:10.1038/s41467-020-15002-8)
Supplement: Supplementary file 11 — Reporting Summary [file 41467_2020_15002_MOESM11_ESM.pdf]

nature research

Corresponding author(s): Heidi Hehnly

Last updated by author(s): Jan 28, 2020

Reporting Summary

Nature Research wishes to improve the reproducibility of the work that we publish. This form provides structure for consistency and transparency in reporting. For further information on Nature Research policies, see Authors & Referees and the Editorial Policy Checklist.

Statistics

For all statistical analyses, confirm that the following items are present in the figure legend, table legend, main text, or Methods section.

n/a	Confirmed	
☐	☒	The exact sample size (*n*) for each experimental group/condition, given as a discrete number and unit of measurement
☐	☒	A statement on whether measurements were taken from distinct samples or whether the same sample was measured repeatedly
☐	☒	The statistical test(s) used AND whether they are one- or two-sided<br>*Only common tests should be described solely by name; describe more complex techniques in the Methods section.*
☐	☒	A description of all covariates tested
☐	☒	A description of any assumptions or corrections, such as tests of normality and adjustment for multiple comparisons
☐	☒	A full description of the statistical parameters including central tendency (e.g. means) or other basic estimates (e.g. regression coefficient) AND variation (e.g. standard deviation) or associated estimates of uncertainty (e.g. confidence intervals)
☐	☒	For null hypothesis testing, the test statistic (e.g. *F*, *t*, *r*) with confidence intervals, effect sizes, degrees of freedom and *P* value noted<br>*Give P values as exact values whenever suitable.*
☒	☐	For Bayesian analysis, information on the choice of priors and Markov chain Monte Carlo settings
☒	☐	For hierarchical and complex designs, identification of the appropriate level for tests and full reporting of outcomes
☒	☐	Estimates of effect sizes (e.g. Cohen's *d*, Pearson's *r*), indicating how they were calculated

Our web collection on statistics for biologists contains articles on many of the points above.

Software and code

Policy information about availability of computer code

Data collection	Volocity Acquisition software (Perkin-Elmer), LASX (Leica), VisiView (Visitron)
Data analysis	GraphPad Prism, ImageJ/FIJI, Imaris (Bitplane), SVI Huygens deconvolution software

For manuscripts utilizing custom algorithms or software that are central to the research but not yet described in published literature, software must be made available to editors/reviewers. We strongly encourage code deposition in a community repository (e.g. GitHub). See the Nature Research guidelines for submitting code & software for further information.

Data

Policy information about availability of data

All manuscripts must include a data availability statement. This statement should provide the following information, where applicable:

- Accession codes, unique identifiers, or web links for publicly available datasets
- A list of figures that have associated raw data
- A description of any restrictions on data availability

The source data underlying all graphs in this study have been provided in a Source Data file.

Field-specific reporting

Please select the one below that is the best fit for your research. If you are not sure, read the appropriate sections before making your selection.

☒ Life sciences ☐ Behavioural & social sciences ☐ Ecological, evolutionary & environmental sciences

For a reference copy of the document with all sections, see nature.com/documents/nr-reporting-summary-flat.pdf

Life sciences study design

All studies must disclose on these points even when the disclosure is negative.

Sample size	Sample size chosen was based on previous Kupffer's vesicle studies (Dasgupta et al 2018 eLife, Wang et al 2012 Dev. Bio.). At least 2-3 embryos were analyzed from n>3 independent clutches of embryos for all experiments.
Data exclusions	No data is excluded from the analysis.
Replication	All studies were easily reproducible over n of 3 experiments.
Randomization	Embryos were not selected based on any particular trait and clutches were divided evenly for treatments performed.
Blinding	Blinding was not feasible for all experiments. Location of mRNA expression needed to be taken in to account for optogenetic treatments in order to determine treatment group.

Reporting for specific materials, systems and methods

We require information from authors about some types of materials, experimental systems and methods used in many studies. Here, indicate whether each material, system or method listed is relevant to your study. If you are not sure if a list item applies to your research, read the appropriate section before selecting a response.

Materials & experimental systems

n/a	Involved in the study	
☐	☒	Antibodies
☐	☒	Eukaryotic cell lines
☒	☐	Palaeontology
☐	☒	Animals and other organisms
☒	☐	Human research participants
☒	☐	Clinical data

Methods

n/a	Involved in the study	
☒	☐	ChIP-seq
☒	☐	Flow cytometry
☒	☐	MRI-based neuroimaging

Antibodies

Antibodies used	Anti-GFP (1:200, Invitrogen A-11122); Phospho-Histone 3 (ser10) (1:200, Cell Signaling 9701S); MKLP1 (N-19) (1:200, Santa Cruz sc-867); aPKC (zeta) (1:200, Santa Cruz sc-216); RacGAP (1:200, Abcam ab2270); alpha-tubulin conjugated with FITC (1:200, Sigma Aldrich F2168); PLK1 (1:200, Cell Signaling Technology 4513S); Acetylated tubulin (1:200, Sigma Aldrich 45-T6793); ZO-1 (1:200, Invitrogen 484333A); Rab11 (1:500, Cell Signaling Technology 3539S); GAPDH-HRP (1:40000, Sigma Aldrich (45-G9295); Secondary: Donkey-anti-Mouse or -Rabbit in 488, 568, 647 (1:200, Life Technologies A21202, A21206, A10042, A10037, A31571, A31573)
Validation	All antibodies were validated based on published localization in tissue culture or zebrafish settings, or compared to live protein expression localization. Catalog numbers are provided for each antibody as well, and each antibody has been independently validated through the manufacturer and has been published as well in tissue culture or zebrafish.

Eukaryotic cell lines

Policy information about cell lines

Cell line source(s)	HeLa cells supplied through ATCC (CCL-2), zebrafish lines supplied from Bagnat Lab at Duke University (CFTR-GFP) and Amack Lab at SUNY Upstate (Sox17:GFP-CAAX, Sox17:GFP, Sox17:dsRed, TAB/wild-type)
Authentication	HeLa cells are authenticated using STR profiling through ATCC when needed, zebrafish lines are authenticated thorugh genotyping and reporter verification.
Mycoplasma contamination	Mycoplasma kit (PlasmoTest, Invivogen) is used in the lab when needed.
Commonly misidentified lines (See ICLAC register)	HeLa cells are authenticated using STR profiling through ATCC when needed.

Animals and other organisms

Policy information about studies involving animals; ARRIVE guidelines recommended for reporting animal research

Laboratory animals	Zebrafish strains used include: TAB (wild-type, Zebrafish International Resource Center), Tg(sox17:GFP-CAAX)sny101 (Dasgupta

Laboratory animals	et al 2018), tgBAC(CFTR-GFP) (Navis et al 2013), Tg(sox17:GFP) (Sakaguchi et al 2006), Tg(sox17:dsRed) (Chung et al 2008). Adult males and females (3 months-1.5 years of age) were used from each line to produce embryos.
Wild animals	No wild animals were utilized in this study.
Field-collected samples	No field-collected samples were utilized in this study.
Ethics oversight	Animal protocol: #18-006 approved by Syracuse University IACUC committee

Note that full information on the approval of the study protocol must also be provided in the manuscript.
